# Metabolic profiling reveals potential biomarkers and underlying signaling pathways involved in mindfulness-based cognitive therapy-improved adolescent depression symptoms

**DOI:** 10.17179/excli2025-8918

**Published:** 2026-01-05

**Authors:** Chun-Hua Xu, Bi-Lan Zhang, Chun-Lan Guan, Lin Wang, Shan Chao, He Li, Qiu-Ping Wu, Da-Jin Zhou, Guo-Qing Min, Fan Yang

**Affiliations:** 1 Department of Children and Adolescents, Anding Hospital Wuhu Hospital (Wuhu Fourth People's Hospital), Wuhu, China; 2 Department of Nursing, Anding Hospital Wuhu Hospital (Wuhu Fourth People's Hospital), Wuhu, China; 3 Department of Psychology, Anding Hospital Wuhu Hospital (Wuhu Fourth People's Hospital), Wuhu, China; 4 The Research Center for Lin He Academician New Medicine, Institutes for Shanghai Pudong Decoding Life, Shanghai, China; 5 Lishui Key Laboratory of Brain Health and Severe Brain Disorders, Lishui Second People's Hospital Affiliated to Wenzhou Medical University, Lishui, China; 6 Department of Children and Adolescents, Lishui Second People's Hospital Affiliated to Wenzhou Medical University, Lishui, China; 7 Department of Clinical Laboratory, Lishui Second People's Hospital Affiliated to Wenzhou Medical University, Lishui, China; 8 Bio-X Institutes, Key Laboratory for the Genetics of Developmental and Neuropsychiatric Disorders, Ministry of Education, Shanghai Jiao Tong University, Shanghai, China

**Keywords:** mindfulness-based cognitive therapy, adolescent depression, untargeted metabolomics, differentially abundant metabolite, biomarker, metabolic regulation pathway

## Abstract

Mindfulness-based cognitive therapy (MBCT) demonstrates significant efficacy in improving depressive symptoms and modulating metabolic profiles. However, the specific metabolite biomarkers and metabolic pathways underlying MBCT's therapeutic effects in adolescent depression remain unclear. This study aims to identify potential metabolite biomarkers and metabolic regulation pathways associated with MBCT improvement of adolescent depression. A global untargeted metabolomics approach was employed to analyze plasma samples from 35 adolescents with depression undergoing MBCT, 35 receiving conventional treatment (CT), and 30 age- and sex-matched healthy controls. MBCT significantly alleviated anxiety and depression symptoms of adolescent patients visualized by SDS, GAD-7, and SCL-90 scores (*P* < 0.0001). Untargeted metabolomics analysis revealed distinct metabolic profile changes in MBCT group compared to CT group, with 203 metabolites significantly upregulated and 186 significantly downregulated in MBCT group (*P* < 0.05). Notably, circulating levels of metabolites such as 10,11-epoxy-3-geranylgeranylindole and paspalicine showed marked increases (*P* < 0.05), whereas abundances of arachidonic acid and L-glutamic acid exhibited significant decreases (*P* < 0.05). KEGG pathway enrichment analysis indicated that the 186 downregulated metabolites were primarily enriched in pathways such as long-term depression, synaptic vesicle cycle, GnRH signaling, and aspartate and glutamate metabolism. Pearson's correlation analysis suggested that arachidonic acid level changes was significantly correlated with clinical improvement of SDS and SCL-90 scores (adjusted* P* < 0.05). ROC analysis revealed that a combination of five metabolites, including 10,11-epoxy-3-geranylgeranylindole, (1S,2R)-1-C-(indol-3-yl) glycerol 3-phosphate, paspalicine, FO 2546E, and FO 2546M, exhibited strong predictive potential for MBCT efficacy (AUC = 0.9061). These findings suggested that arachidonic acid involved in the long-term depression pathway may play pivotal roles in MBCT improvement of adolescent depression. This study provides insight into the potential biomarkers and metabolic regulation mechanisms underlying MBCT's therapeutic effects and theoretical guidance for clinical practice in MBCT intervention for adolescent depression.

See also the graphical abstract(Fig. 1).

## Abbreviations

APWD: Adolescent Patients with Depression

AUC: Area Under the Curve

BH: Benjamini-Hochberg

BPC: Base Peak Chromatogram

CT: Conventional Treatment

DAM: Differentially Abundant Metabolite

DSM-5: The Diagnostic and Statistical Manual of Mental Disorders, Fifth Edition

GAD-7: Generalized Anxiety Disorder-7

HC: Healthy Control

ICD-11: International Classification of Diseases, 11^th^ Revision

KEGG: Kyoto Encyclopedia of Genes and Genomes

K-SADS: Kiddie Schedule for Affective Disorders and Schizophrenia

MBCT: Mindfulness-based Cognitive Therapy

MDD: Major Depressive Disorder

OPLS-DA: Orthogonal Partial Least Squares Discriminant Analysis

PCA: Principal Component Analysis

PLS-DA: Partial Least Squares-Discriminant Analysis

ROC: Receiver Operator Characteristic

RSD: Relative Standard Deviation

SCL-90: Symptom Checklist-90

SDS: Self-rating Depression Scale

VIP: Variable Importance for the Projection

## 1 Introduction

Depression, a widespread mental disorder with significant global health burden, affects over 264 million people worldwide and is a leading cause of disability (Malhi and Mann, 2018[71], Stecher et al., 2024[112]). In China, approximately 54 million individuals are impacted (Huang et al., 2019[52]). The economic burden of depression is significant, involving direct healthcare costs, productivity loss, and increased morbidity and mortality (Stecher et al., 2024[112]). Traditional treatments like antidepressants, electroconvulsive therapy (ECT), and transcranial magnetic stimulation (TMS) are effective but often have side effects and variable responses (Rush et al., 2006[99]). Adolescent depression, a pressing public health issue with high prevalence and severe consequences, affects 15 % to 30 % of Chinese adolescents (Rao et al., 2019[91][92], Tang et al., 2019[116]). Adolescent depression's etiology is multifactorial, involving genetic, environmental, and psychosocial factors (Furukawa, 2020[34], Diener et al., 2021[25]). The lack of reliable biomarkers complicates diagnosis and treatment. Metabolic pathway disruptions play a crucial role in its development, with altered metabolic profiles linked to depressive symptoms (Holmes et al., 2008[49]).

Mindfulness-based cognitive therapy (MBCT), a non-drug and non-invasive therapy integrating cognitive-behavioral therapy with mindfulness practices, was developed by Zindel Segal et al. (Morgan, 2003[79], Williams and Kuyken, 2012[133]). It shows promise in treating mental disorders, especially in preventing relapse in recurrent major depressive disorder (MDD) (Teasdale et al., 2000[117], Evans et al., 2008[27], Barnhofer et al., 2009[8], Weber et al., 2010[130], Chiesa and Serretti, 2011[16], Deckersbach et al., 2012[24], Howells et al., 2012[51], Williams and Kuyken, 2012[133], Gu et al., 2015[42], Segal and Walsh, 2016[104], Wong et al., 2016[135], Cladder-Micus et al., 2018[17], Lovas and Schuman-Olivier, 2018[69], Ter Avest et al., 2019[118], Torres-Platas et al., 2019[123], Zhao et al., 2019[146], Chan et al., 2020[14], Ford et al., 2020[30], Segal et al., 2020[103], Xuan et al., 2020[139], Fattahi et al., 2021[28], McCartney et al., 2021[76], Jiang et al., 2022[54], Zhang et al., 2022[145], Cladder-Micus et al., 2023[18], Sverre et al., 2023[115], Weintraub et al., 2023[131], Mak et al., 2024[70]). Recent studies highlight its efficacy in reducing symptoms and preventing relapse (Piet and Hougaard, 2011[88], Williams et al., 2014[132], Davidson, 2016[20], Kuyken et al., 2016[61], Riemann et al., 2016[95], Shawyer et al., 2016[105], Meadows and Shawyer, 2017[78], Musa et al., 2021[81], de Klerk-Sluis et al., 2022[21], Jakary et al., 2023[53], Liu et al., 2024[67]). The mechanisms of MBCT's therapeutic effects are still not well understood, warranting more research into its biological and metabolic basis (van der Velden et al., 2015[125]). Studies suggest MBCT may improve depressive symptoms by modulating metabolic profiles and pathways (Gex-Fabry et al., 2012[37], Li et al., 2016[66], Wang et al., 2022[128]). For example, Li et al. found significant metabolite differences in MDD patients after MBCT, linked to depression severity improvement (Li et al., 2016[66]). Wang et al. showed imbalanced microbiota and their metabolites might enhance MBCT's effectiveness via the microbiota-gut-brain axis (Wang et al., 2022[128]). However, the molecular mechanisms of MBCT's influence on metabolic pathways to alleviate symptoms remain largely unexplored. Clinically, the lack of sensitive and specific biomarkers hinders assessing MBCT's efficacy and prognosis. Identifying such biomarkers is crucial for advancing MBCT's clinical application and improving outcomes. While many studies show MBCT improves mental disorder symptoms, and some clarify how MBCT alters metabolic profiles and pathways, its effects on adolescent depressive symptoms and impact on the adolescent depression metabolic characteristics remain largely unknown. In this study, we used a global untargeted metabolomics approach to analyze plasma samples from adolescent depression patients and age- and sex-matched healthy controls. We compared metabolic changes in adolescent patients after MBCT and conventional treatment, identified potential metabolites and pathways influenced by MBCT, and found several potential biomarkers for predicting MBCT efficacy.

Our results further reveal the plasma metabolic profile characteristics of adolescent depression patients and identify candidate metabolites and metabolic signaling pathways involved in MBCT's improvement of depressive symptoms. This deeper understanding of MBCT's metabolic regulatory mechanisms in treating adolescent depression helps identify potential biomarkers for predicting MBCT efficacy. To our knowledge, this is the first study to investigate metabolic profile changes and potential signaling pathways in adolescent depression patients after MBCT treatment, preliminarily revealing MBCT's metabolic regulatory mechanisms and candidate biomarkers in treating adolescent depression. This study has significant theoretical and clinical implications for applying MBCT in treating adolescent depression.

## 2 Materials and Methods

### 2.1 Subject recruitment

A total of 70 adolescent patients with depression, diagnosed according to the DSM-5 and ICD-11 criteria, were recruited from Wuhu, Anhui Province, China, and randomly assigned to two groups of 35 patients each: one group received conventional treatment (CT), while the other group underwent mindfulness-based cognitive therapy (MBCT) in addition to CT. The process of random allocation includes random sequence generation using a computerized random number generator and allocation concealment using sequentially numbered opaque envelopes, and blinding step was conducted by using independent assessors who were unaware of the participants' group assignments. Additionally, 30 age- and sex-matched mentally healthy participants were recruited as healthy controls for metabolomics analysis. Demographic information for all subjects is presented in Table 1(Tab. 1). The study protocols were reviewed and approved by the Ethics Committee of Wuhu Fourth People's Hospital (Approval number [2022]--KY--14). Written informed consent was obtained from each participant or their legal guardian prior to recruitment. Inclusion criteria for adolescent patients with depression are as follows: aged 11-20 years; Meet the diagnostic criteria for major depressive disorder (MDD) according to the DSM-5 or ICD-11, confirmed by a structured clinical interview (e.g., K-SADS); Not currently on antidepressant medication within the past 6 months; Elementary education or higher, capable of independently completing questionnaires; Both the subject and legal guardian are willing to participate in the study, with written informed consent provided by the participant and legal guardian. Inclusion criteria for healthy controls are as follows: aged 11-20 years; No personal or family history of psychiatric disorders, confirmed by clinical interview; No severe physical or neurological illnesses; No history of substance abuse or dependence; Not on any psychotropic medication; Written informed consent provided by participant and parent/guardian. Exclusion criteria for both groups are as follows: History of neurological disorders (e.g., epilepsy, traumatic brain injury), severe physical illnesses (e.g., cardiovascular, respiratory, or metabolic disorders), or substance abuse/dependence within 6 months; Severe somatic illnesses that could confound depression assessment or treatment; Presence of language or communication disorders; Exposure to severe traumatic events within the past 3 months; Use of medications known to significantly affect metabolism or mental state within the past 6 months; Unwillingness or inability to comply with study protocols, including attendance at therapy sessions and metabolomics sampling; Refusal to sign the written informed consent; Subjects who withdrew from the study; For metabolomics analysis, exclusion of participants who have had significant changes in diet, exercise, or supplementation within 72 hours prior to blood sampling.

### 2.2 MBCT and CT intervention

All participants in the CT group received standard care, psychological support, and health education. In addition to CT intervention, subjects in the MBCT group underwent MBCT. The MBCT procedure is as follows: Week 1: Introduce the origins, concepts, and efficacy of Mindfulness-Based Stress Reduction (MBSR). Begin with the raisin exercise: Guide patients to relax, place a raisin in the palm, and observe it as if encountering it for the first time. Encourage exploration through sight, touch, smell, sound, taste, and mindful awareness of the entire process from ingestion to digestion. This practice cultivates focus, calm, and attention; Week 2: Practice mindful breathing and mountain meditation. Patients sit or lie down, relax the body, and focus on their breath, redirecting attention to breathing if distracted. Visualize oneself as a mountain, with arms as slopes and head as the peak, experiencing seasonal changes (spring, summer, autumn, winter) while remaining steadfast. Patients are instructed to avoid judgment or analysis, fostering an internal sense of stability and calm. This practice encourages resilience in facing physical and emotional discomfort; Week 3: Introduce mindful yoga and mindful walking. Transition from static to dynamic exercises, using stretching and mindful movement to alter habitual patterns. This enhances focus, deepens body-mind awareness, and integrates mindfulness into daily activities; Week 4: Practice body scan and loving-kindness blessings. Patients sit or lie down with gentle music, systematically relaxing each body part from head to toe while cultivating mindful awareness. After relaxation, patients extend heartfelt blessings to themselves, loved ones, acquaintances, those in distress, and all beings. This practice encourages forgiveness, self-compassion, and emotional release, promoting positive behavioral and emotional shifts. Sessions conclude with guided hand rubbing, head/limb movement, and reflection on practice insights. The GAD-7, SDS, and SCL-90 scales were administered to all participants pre- and post-intervention to assess the amelioration of anxiety and depressive symptoms following MBCT or CT.

### 2.3 Biospecimen collection

Approximately 2.0 mL of peripheral blood samples were collected from all patients and healthy subjects using EDTA-anticoagulant vacuum blood collection tubes. The mixture was gently mixed by inverting it six to ten times to ensure that the blood would not clot. Subsequently, blood samples were centrifuged at 3,500 rpm for 10 minutes at 4 °C, and the plasma was transferred to new centrifuge tubes and quickly frozen in liquid nitrogen. Immediately, all separated plasma was stored at -80 °C for future LC-MS/MS analysis.

### 2.4 LC-MS/MS-based global untargeted metabolomics analysis

#### 2.4.1 Extraction of metabolites

All samples were thawed at 4 °C and vortexed for 60 seconds. A volume of 100 μL from each sample was transferred to new 2 mL tubes. Next, 400 μL of methanol solution (precooled at -20 °C) were added to each tube. The samples were thoroughly vortexed for 60 seconds. Following this, the samples were centrifuged at 12,000 rpm and 4 °C for 10 minutes. After centrifugation, 500 μL of supernatant from each sample was pipetted to another 2 mL tube. The samples were then dried under vacuum. Once dry, the samples were dissolved in 150 μL of 2-chloro-phenylalanine (4 ppm) solution prepared with 80 % methanol water, then were filtered by 0.22 μm membrane and transferred into the detection bottle for LC-MS analysis. Quality control (QC) samples were used to monitor any deviations in the analytical results (Sangster et al., 2006[102], Zelena et al., 2009[144]).

#### 2.4.2 Liquid chromatographic assay

Chromatographic separation assay was performed on a Vanquish UHPLC System (ThermoFisher Scientific, USA) coupled with an ACQUITY UPLC® HSS T3 column (2.1 × 100 mm, 1.8 μm) (Waters Co. Ltd., Milford, MA, USA) (Yang et al., 2021[140]). The temperature of column was set to 40 °C. The flow rate and injection volume were set at 0.3 mL/minutes and 2.0 μL, respectively. For LC-ESI (+)-MS analysis, the mobile phases consisted of (B2) 0.1 % formic acid in acetonitrile (v/v) and (A2) 0.1 % formic acid in water (v/v), gradient elution of analytes was carried out with the following steps: 0~1.0 minutes, 10 % B2; 1.0~5.0 minutes, 10 %~98 % B2; 5.0~6.5 minutes, 98 % B2; 6.5~6.6 minutes, 98 %~10 % B2; 6.6~8.0 minutes, 10 % B2. For LC-ESI (-)-MS analysis, the mobile phases consisted of (B3) acetonitrile and (A3) ammonium formate (5 mM), gradient elution of analytes was carried out with the following steps: 0~1.0 minutes, 10 % B3; 1.0~5.0 minutes, 10 %~98 % B3; 5.0~6.5 minutes, 98 % B3; 6.5~6.6 minutes, 98 %~10 % B3; 6.6~8.0 minutes, 10 % B3 (Zelena et al., 2009[144]).

#### 2.4.3 Mass spectrometry analysis

Detection of metabolites was performed on Orbitrap Exploris 120 spectrometer (ThermoFisher Scientific, USA) with ESI ion source (both positive and negative model). Simultaneous MS1 and MS/MS (full MS mode with data-dependent acquisition, DDA) was employed. The parameters were as follows: sheath gases pressure, 40 arbitrary (arb) units, auxiliary gases pressure, 10 arb; spray voltage, 3.50 kV for ESI (+), and -2.50 kV for ESI (-); capillary temperature, 325 °C; MS1 scan range, mass/charge (m/z) 100-1000; MS1 resolving power, 60,000 FWHM; number of data dependent scans per cycle, 4; MS/MS resolving power, 15,000 FWHM; normalized collision energy, 30 %; dynamic exclusion time, automatic (Want et al., 2013[129]).

#### 2.4.4 Data acquisition

Chromatography was employed for component separation, with subsequent data acquisition performed via mass spectrometry (MS). Each scan generated a mass spectrum, where the most intense ion was continuously tracked. Plotting ion intensity on the y-axis against time on the x-axis yielded what is known as the base peak chromatogram (BPC). High-accuracy MS data were recorded using the MassLynx 4.1 software (Waters Co. Ltd., Milford, MA, USA).

#### 2.4.5 Data processing

Raw data were converted to mzXML format using ProteoWizard software (v3.0.8789) (Rasmussen et al., 2022[93]). Peak identification, filtration, and alignment were performed with the XCMS R package (v.3.12.0) (Navarro-Reig et al., 2015[82]). The resulting data matrix included m/z ratios, retention times, and relative peak area ratios. A total of 8,814 and 9,749 precursor ions were detected in the positive and negative ion mode, respectively.

#### 2.4.6 Data quality control

For biomarker identification, the relative standard deviation (RSD) of a characteristic peak in the QC sample must be within 30 %. Peaks exceeding this threshold were excluded. This process enhances dataset quality by removing poorly reproducible peaks, facilitating more reliable biomarker detection. Our QC samples showed that approximately 70 % of peaks had RSD values below 30 %, indicating acceptable data quality. A total of 2,718 positive ions and 2,805 negative ions were filtered out, and 6,096 positive ions and 6,944 negative ions remained for downstream analysis.

#### 2.4.7 Data normalization

To stabilize data variance, reduce the impact of extreme values, and allow for the retention of information from samples with low metabolite levels, we performed data normalization using the SIMCA-P (v.14.1) (Wu et al., 2025[136]) with the following parameters: Distance to model (normalized in units of standard deviation, weighted by the modeling power), Coefficients (scaled and centered), Residuals/R2 (standardized), Select the type of R2 (R2-explained variation). A Log2 transformation to the raw data was applied to ensure the robustness of metabolomics analysis. The transformed data were used for downstream analyses.

#### 2.4.8 Confounding factor correction

To address the potential confounding effects of age and sex on the metabolome data, we performed a series of data preprocessing steps. Initially, we used a linear regression model to evaluate the impact of age on metabolite levels. This analysis helped us to identify age-related variations in the metabolic profiles. Following this, we incorporated age as a covariate in our statistical models to adjust for its influence on metabolite abundance. For sex-related differences, we also applied a linear regression model to assess the association between metabolite levels and sex. This step allowed us to control for sex differences when estimating the relationship between metabolite abundance and the study outcomes. The regression models were applied to each metabolite in the dataset to obtain adjusted abundance estimates. To correct for batch effects, we first conducted a principal component analysis (PCA) to visualize and identify any systematic variations in the dataset. Subsequently, we utilized the ComBat method from the SVA package (v.3.42.0) (Leek et al., 2012[64]) in R to adjust for batch effects. The ComBat method effectively removes batch-related variations while preserving the biological variability in the data. After completing these adjustment steps, the data were used for all downstream analyses to ensure that the results were not influenced by batch or demographic confounders.

### 2.5 Bioinformatic analysis

#### 2.5.1 Agglomerate hierarchical clustering

We utilized agglomerative hierarchical clustering to group objects into increasingly larger classes. Metabolite relative quantification was performed via the Pheatmap package (v.4.3.3) in R (Gatto et al., 2015[36]). Sample and data analysis involved distance-based matrix calculations, with clustering achieved through the average-linkage method.

#### 2.5.2 Multivariate statistical analysis

Prior to conducting multivariate statistical analysis, metabolomic data are often transformed through auto-scaling to enhance interpretability and reliability. In this study, we applied autoscaling prior to multivariate analysis to optimize result clarity. The analysis was performed using the Ropls package (v.1.22.0) in R, which included both unsupervised techniques like PCA and supervised methods such as partial least squares-discriminant analysis (PLS-DA) and orthogonal PLS-DA (OPLS-DA) (Rohart et al., 2017[98], Yang et al., 2024[141]). These methods were selected to provide comprehensive insights into the metabolic data structure and patterns.

#### 2.5.3 Identification of differentially abundant metabolites

Differentially abundant metabolites were identified using variable importance for the projection (VIP) scores exceeding 1.00 and P values below 0.05. The VIP values were calculated using MetaboAnalyst 4.0 (www.metaboanalyst.ca) and R package (v.4.3.3). Metabolites identification involved verifying molecular weights with errors within 15 ppm, followed by matching and annotating against multiple databases, including Human Metabolome Database (HMDB) (http://www.hmdb.ca) (Wishart et al., 2007[134]), Metlin (https://metlin.scripps.edu), Lipid Maps (http://www.lipidmaps.org) (Sud et al., 2007[113]), mzClound (http://www.mzcloud.org) (Abdelrazig et al., 2020[1]), MassBank (http://massbank.jp) (Horai et al., 2010[50]), and MoNA (https://mona.fiehnlab.ucdavis.edu). A custom database, built by BioNovoGene Co., Ltd. (Suzhou, China) using MS/MS fragment data, was also utilized. Identified metabolites were classified via Kyoto Encyclopedia of Genes and Genomes (KEGG) (Ogata et al., 1999[85]) and Metabolon databases. For differential analysis, Z-scores were calculated based on metabolite levels relative to control group means and standard deviations, transforming data for standardized comparison, which was used to measure the relative content of metabolites at the same level. Agglomerative hierarchical clustering generated heatmaps, and Pearson correlations were computed using the cor() package (v.4.0.3) in R to analyze the correlation between metabolites, with significance at P < 0.05. The pROC package (v.1.18.0) (Robin et al., 2011[97]) in R was used to perform receiver operator characteristic (ROC) analysis, ROC analysis assessed potential biomarker performance through area under the curve (AUC). MetPA within MetaboAnalyst 4.0 (www.metaboanalyst.ca) analyzed metabolic pathways using hypergeometric tests, with pathway topology based on betweenness centrality. Relative pathway response values were derived from metabolite data and dimensionality reduction, enabling pathway correlation analysis and network visualization.

### 2.6 Statistical analysis methods

Shapiro-Wilk test was used to examine the normal distribution of data (such as age, GAD-7, SDS, and SCL-90 score) from each group. Levene test was used to evaluate homogeneity of variance across groups. One-way ANOVA and Tukey's multiple comparisons post-hoc test was used to examine the statistical differences of age among the three groups (namely MBCT, CT, and HC). Chi-square test was used to examine the statistical differences of sex among the three groups (i.e., MBCT, CT, and HC). If data was normally distributed, paired t-test was used to examine the statistical differences of GAD-7, SDS or SCL-90 score between pre- and post-intervention in MBCT or CT group, otherwise Wilcoxon Signed-Rank test was used to examine the statistical differences of GAD-7 or SDS score between Pre and Post group in MBCT or CT group. SPSS software V19.0 (Chicago, IL, USA) was used to perform statistical analysis. GraphPad Prism (San Diego, CA, USA) was used to prepare graphs. Benjamini-Hochberg (BH) method was used to correct P values. Two-sided statistical significance was tested, and only an adjusted P (Padj) value < 0.05 was interpreted as statistically significant.

## 3 Results

### 3.1 Mindfulness-based cognitive therapy significantly alleviates positive symptoms in adolescent depression

**Figure 1:**
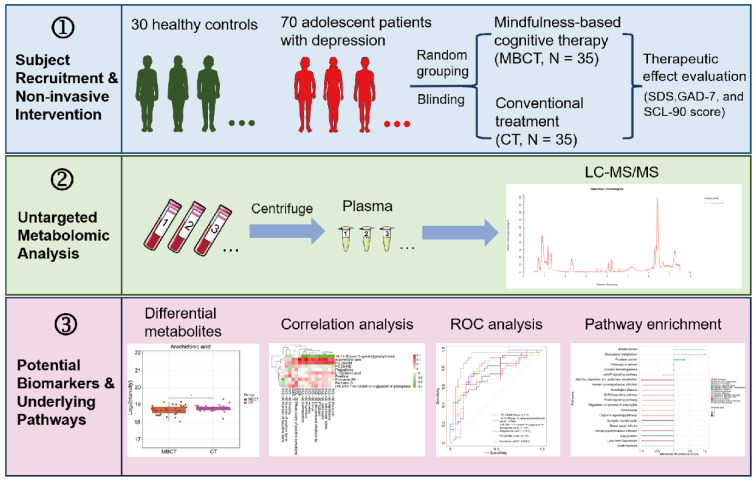
Graphical abstract

A total of 70 adolescent patients with depression (APWD) were recruited and randomly divided into control and observation groups, with 35 patients in each. The control group received conventional treatment (CT), while the observation group received mindfulness-based cognitive therapy (MBCT) in addition to CT. Additionally, 30 age- and sex-matched mentally healthy participants were recruited as healthy controls (HC) for metabolome analysis (Figure 1(Fig. 1)). Patients in the MBCT group had a mean age of 14.83 ± 1.58, including 7 males and 28 females. Those in the CT group had a mean age of 14.69 ± 1.37, with 8 males and 27 females. Participants in the HC group had a mean age of 17.13 ± 1.80, comprising 8 males and 22 females. No significant differences were found in gender distribution among the MBCT, CT, and HC groups (P > 0.05) (Table 1(Tab. 1)). Similarly, no significant age differences were observed between the MBCT and CT groups (P > 0.05) (Table 1(Tab. 1)). However, significant age differences were found between the MBCT and HC groups and between the CT and HC groups (P < 0.001) (Table 1(Tab. 1)).

In the MBCT group, the pre-intervention GAD-7 score of 13.54 ± 4.67, decreasing to 9.03 ± 5.35 post-intervention. This indicates that MBCT significantly reduces anxiety symptoms in adolescents with depression (P < 0.0001) (Table 2(Tab. 2)). In contrast, CT showed no significant improvement in anxiety symptoms (P > 0.05) (Table 2(Tab. 2)). Regarding depression symptoms, the SDS score in the MBCT group decreased from 73.39 ± 10.10 pre-intervention to 61.48 ± 13.59 post-intervention, indicating significant improvement (P < 0.0001) (Table 2(Tab. 2)). Similarly, the CT group's SDS score decreased from 72.95 ± 19.17 pre-intervention to 65.69 ± 14.44 post-intervention, also showing significant improvement (P < 0.0001) (Table 2(Tab. 2)). SCL-90 scale scores revealed significant improvements in psychological symptoms in the MBCT group (P < 0.05) (Table 3(Tab. 3)). Specifically, the total score decreased from 235.40 ± 83.51 pre-intervention to 192.80 ± 87.54 post-intervention, the total mean score decreased from 2.61 ± 0.93 to 2.10 ± 1.01, and the mean score of positive symptoms decreased from 3.56 ± 1.97 to 3.21 ± 3.49. In terms of specific dimensions, the MBCT group demonstrated significant alleviation of positive symptoms (P < 0.05). Somatization scores decreased from 29.03 ± 15.16 to 22.83 ± 13.05, force scores from 27.69 ± 9.91 to 24.26 ± 11.38, interpersonal relationship scores from 25.77 ± 8.92 to 21.31 ± 9.45, depression scores from 39.09 ± 13.48 to 31.54 ± 12.80, anxiety scores from 26.94 ± 10.45 to 22.74 ± 11.78, phobism scores from 17.74 ± 8.75 to 13.51 ± 8.47, paranoid scores from 14.40 ± 6.39 to 11.77 ± 6.58, and psychosis scores from 24.83 ± 9.49 to 19.29 ± 10.32 (Table 3(Tab. 3)). In the CT group, the SCL-90 total score decreased from 262.09 ± 92.01 to 230.09 ± 94.28, and the total mean score decreased from 2.91 ± 1.02 to 2.55 ± 1.05. There were also decreases in interpersonal relationship scores (28.80 ± 9.16 to 25.43 ± 9.54), depression scores (41.83 ± 14.75 to 37.89 ± 14.65), anxiety scores (30.91 ± 11.94 to 26.29 ± 11.60), hostility scores (19.37 ± 9.41 to 15.86 ± 7.01), and phobism scores (28.11 ± 11.69 to 24.31 ± 10.51), indicating partial improvement in positive symptoms (P < 0.05) (Table 3(Tab. 3)). Overall, MBCT intervention significantly improved positive symptoms such as anxiety and depression in adolescent patients with depression. However, the underlying metabolic regulatory mechanisms remain unclear.

### 3.2 Identification of differential metabolites associated with adolescent depression

**Figure 2:**
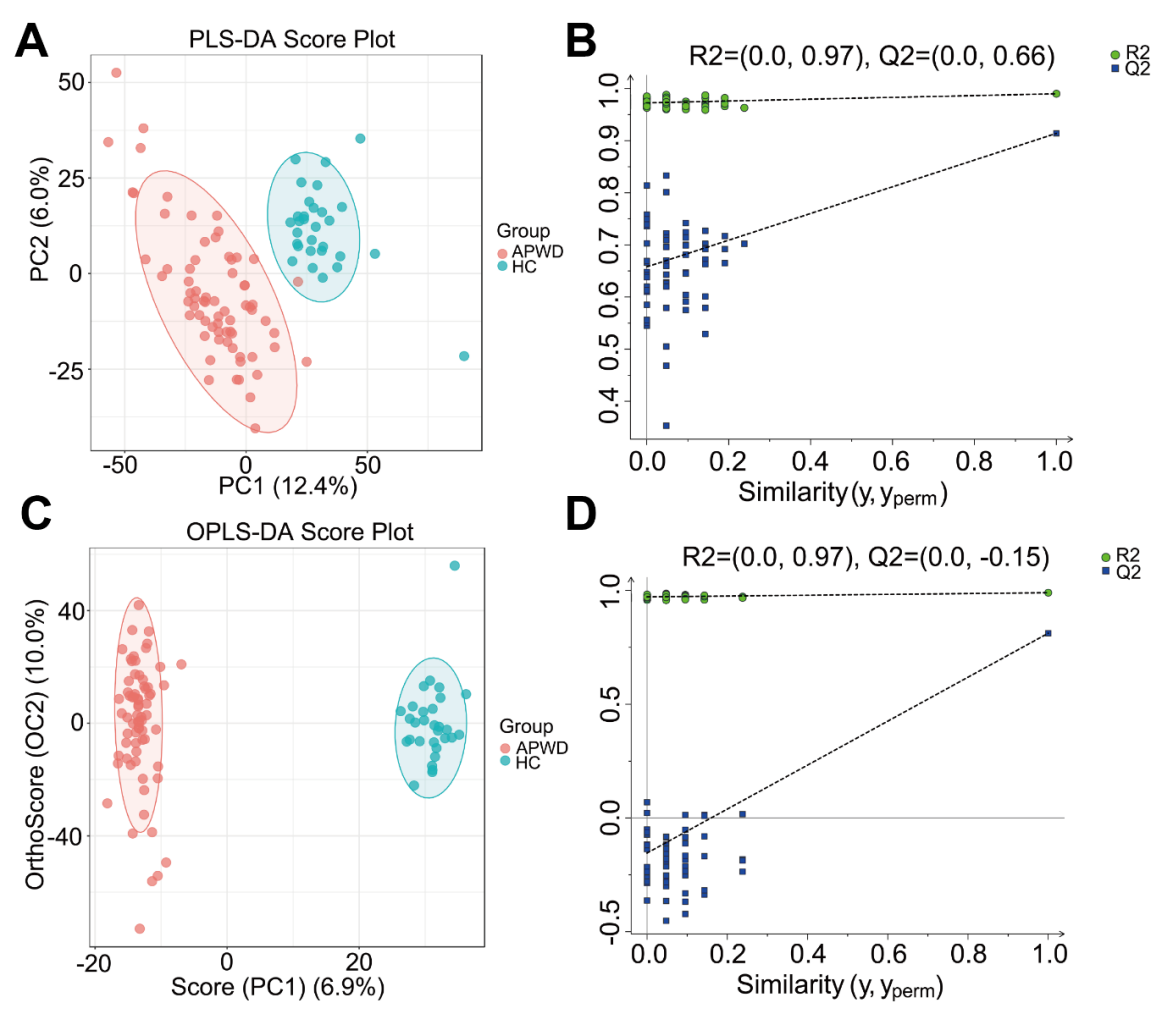
PLS-DA and OPLS-DA models for separating APWD and HC in positive ion mode. (A) and (B) display the PLS-DA plot for the positive ion mode. R2 = (0.0, 0.97), Q2 = (0.0, 0.66). (C) and (D) present the OPLS-DA plot for the positive ion mode. R2 = (0.0, 0.97), Q2 = (0.0, -0.15).

To further investigate the plasma metabolic profiles in APWD receiving MBCT or CT, we employed a global untargeted metabolomics approach based on LC-MS/MS to analyze plasma samples from 70 APWD and 30 HCs (Figure 1(Fig. 1)). We designated the comparative groups as follows: APWD vs. HC (C1), MBCT vs. CT (C2), MBCT vs. HC (C3), and CT vs. HC (C4). In the positive ion mode, supervised analyses including PLS-DA and OPLS-DA revealed a clear separation between adolescent depression samples and HCs (Figures 2A-D(Fig. 2)). Similar patterns were observed in the negative ion mode (Supplementary Figures 1A-D). These results indicated that the plasma metabolic profiles of adolescents with depression were significantly altered compared to those of HC.

**Figure 3:**
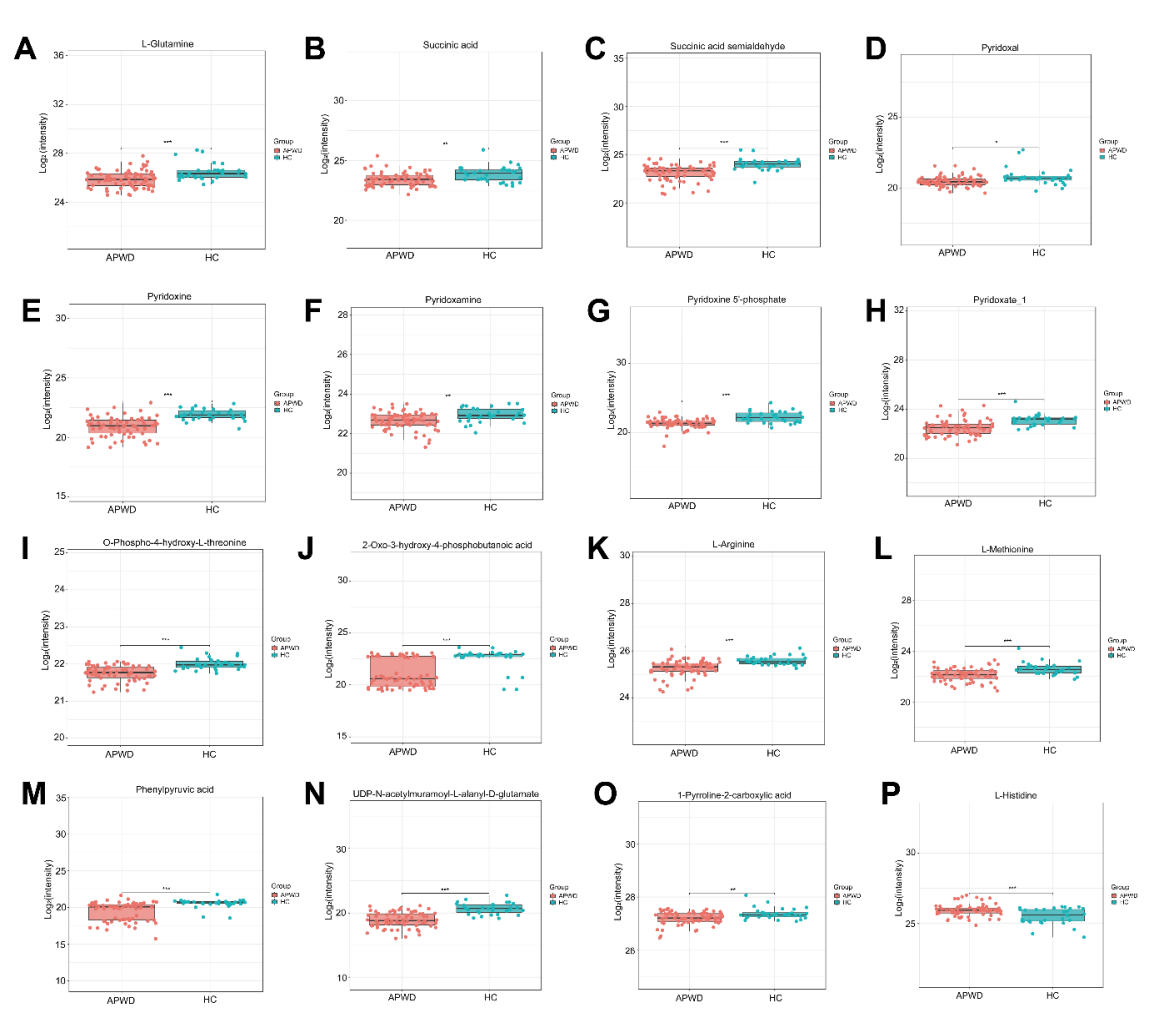
Normalized intensity of sixteen representative DAMs in 70 APWD and 30 HC plasma samples. (A-O) The relative levels of fifteen metabolites, including L-glutamine, succinic acid, succinic acid semialdehyde, pyridoxal, pyridoxine, pyridoxamine, pyridoxine 5'-phosphate, pyridoxate_1, O-phospho-4-hydroxy-L-threonine, 2-oxo-3-hydroxy-4-phosphobutanoic acid, L-arginine, L-methionine, phenylpyruvic acid, UDP-N-acetylmuramoyl-L-alanyl-D-glutamate, and 1-pyrroline-2-carboxylic acid, were significantly decreased in APWD compared to HC, while L-histidine (P) showed a significant increase. Statistical comparisons were performed using a T-test with BH method correction. *: Padj < 0.05, **: Padj < 0.01, ***: Padj < 0.001.

In group C1, heatmap analysis showed distinct differences in the plasma metabolic profiles of adolescents with depression compared to HCs (Supplementary Figure 2A). Differentially abundant metabolites (DAMs) were identified using VIP > 1.0 and P < 0.05 as criteria. A total of 671 DMAs, including 118 up-regulated and 553 down-regulated metabolites, were found in APWD compared to the HC group (Supplementary Figures 2B and C). Among these DAMs, the relative levels of L-glutamine, succinic acid, succinic acid semialdehyde, pyridoxal, pyridoxine, pyridoxamine, pyridoxine 5'-phosphate, pyridoxate_1, O-phospho-4-hydroxy-L-threonine, 2-Oxo-3-hydro-4-phosphobutanoic acid, L-arginine, L-methionine, phenylpyruvic acid, UPD-N-acetylmuramoyl-L-ananyl-D-glutamate and 1-pyrroline-2-carboxylic acid were significantly decreased in APWD compared to HC (adjusted P < 0.05) (Figures 3A-O(Fig. 3)). In contrast, the relative concentration of L-histidine was significantly increased in APWD compared to HC (adjusted P < 0.05) (Figure 3P(Fig. 3)).

**Figure 4:**
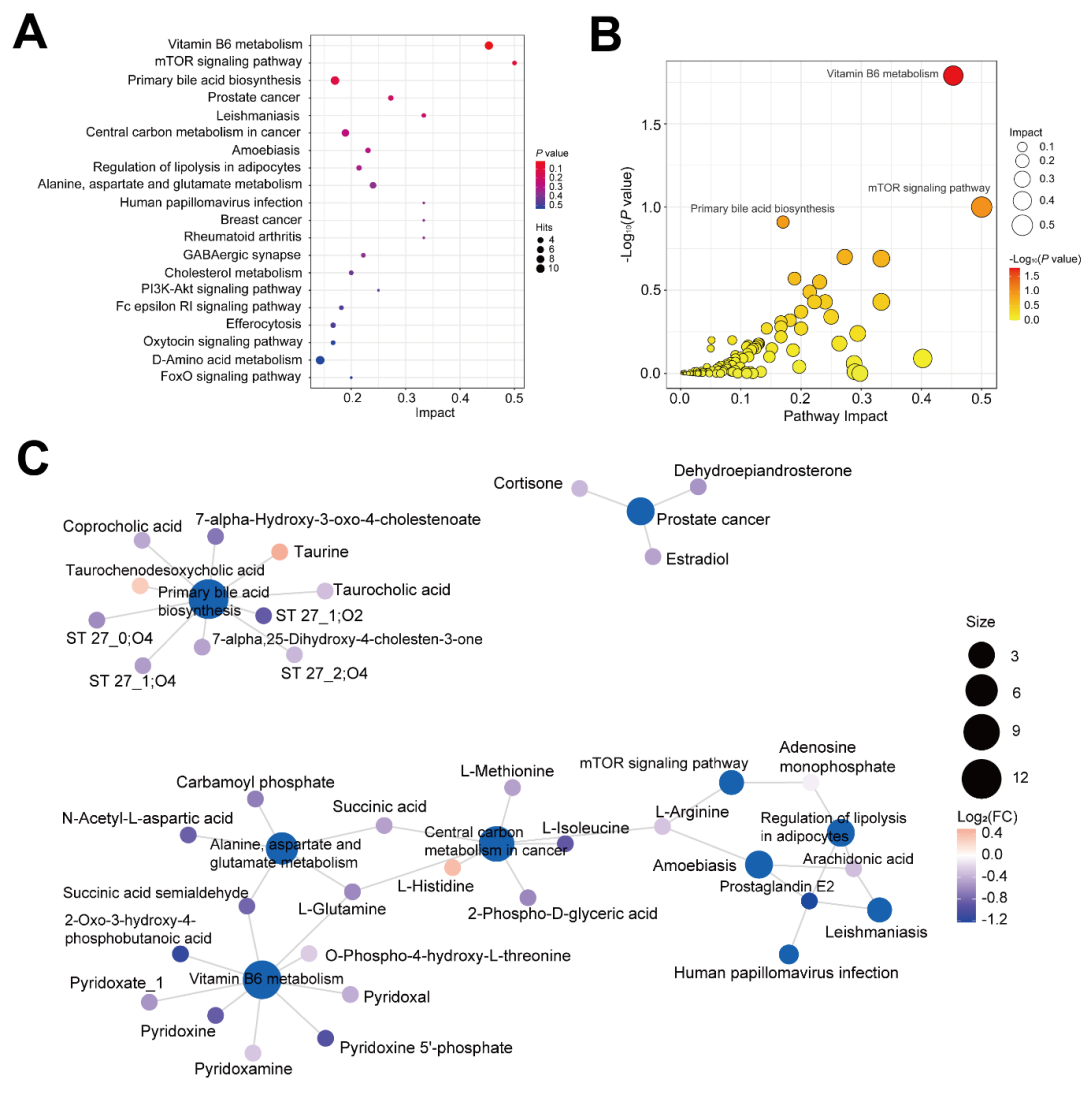
Functional and pathway enrichment analysis of 671 DAMs identified in APWD vs. HC. (A) Top 20 ranking pathways enriched by 671 DAMs. (B) Top-ranking pathways, including vitamin B6 metabolism, mTOR signaling pathway, and primary bile acid biosynthesis, enriched in APWD samples. (C) Interaction network analysis of representative DAMs and their pathways in APWD.

KEGG pathway enrichment analysis revealed that the 671 DMAs were enriched in multiple pathways, including vitamin B6 metabolism, mTOR signaling pathway, primary bile acid biosynthesis, prostate cancer, leishmaniasis, central carbon metabolism in cancer, amoebiasis, regulation of lipolysis in adipocytes, alanine, aspartate and glutamate metabolism, human papillomavirus infection, breast cancer, rheumatoid arthritis, GABAergic synapse, cholesterol metabolism, PI3K-Akt signaling, Fc epsilon RI signaling, efferocytosis, oxytocin signaling, D-amino acid metabolism, as well as FoxO signaling (Figure 4A(Fig. 4)). The most significantly enriched pathway was vitamin B6 metabolism, followed by mTOR signaling and primary bile acid biosynthesis (Figure 4B(Fig. 4)). In addition, pathway interaction network analysis showed that several pathways interacted through key node metabolite. For example, L-glutamine and succinic acid semialdehyde links vitamin B6 metabolism and alanine, aspartate and glutamate metabolism, while L-glutamine and succinic acid connect central carbon metabolism in cancer and alanine, aspartate and glutamate metabolism (Figure 4C(Fig. 4)). Additionally, central carbon metabolism in cancer, mTOR signaling, and amoebiasis interact via L-arginine (Figure 4C(Fig. 4)).

### 3.3 Differential metabolites and underlying signaling pathways associated with MBCT intervention

**Figure 5:**
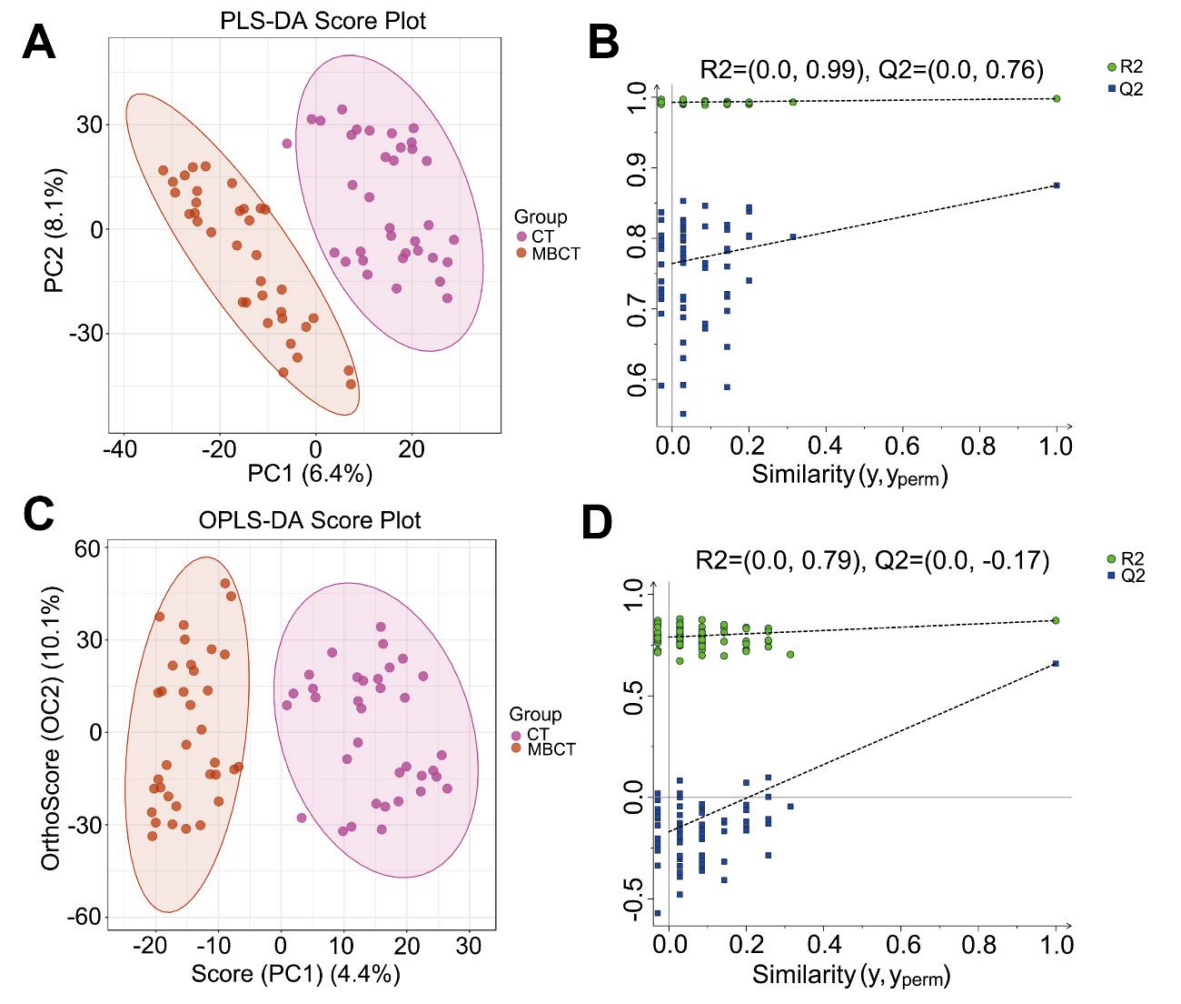
PLS-DA and OPLS-DA models for separating MBCT and CT in positive ion mode. (A) and (B) display the PLS-DA plot for the positive ion mode. R2 = (0.0, 0.99), Q2 = (0.0, 0.76). (C) and (D) present the OPLS-DA plot for the positive ion mode. R2 = (0.0, 0.79), Q2 = (0.0, -0.17).

**Figure 6:**
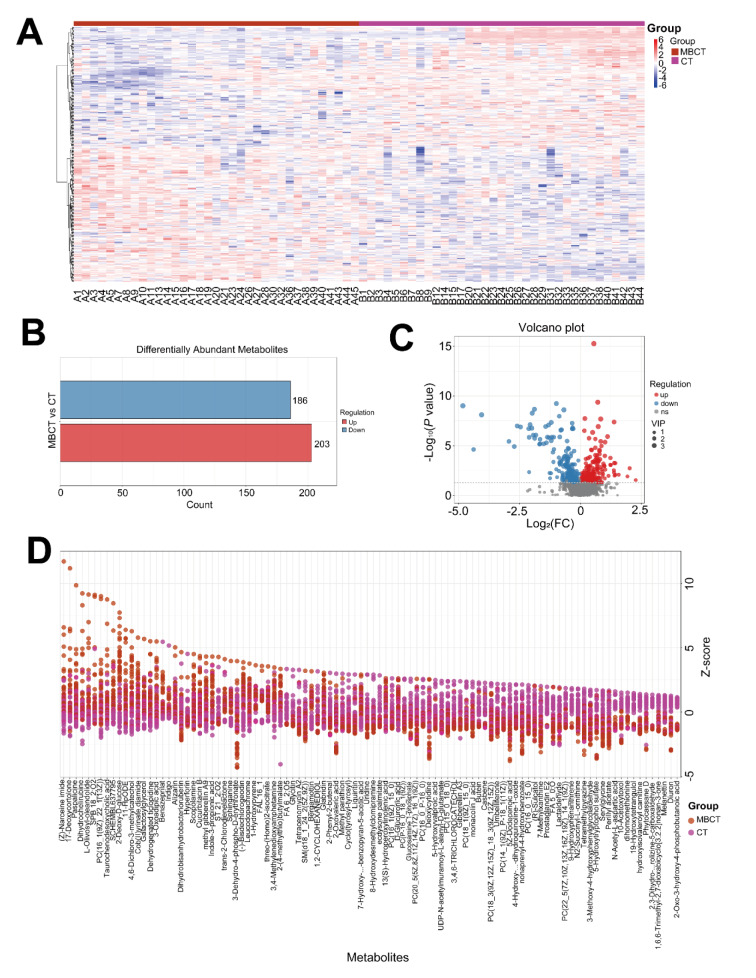
Identification of 389 differential metabolites between MBCT and CT. (A) The heatmap shows 389 DAMs, with blue and red indicating down- and up-regulation, respectively. Relative levels of metabolites were determined by the Pheatmap package (v.4.3.3) in R. (B) The bar plot and (C) the volcano plot illustrates the distribution of DAMs, including 203 up-regulated and 186 down-regulated metabolites in MBCT. (D) Z-score plot of concentration changes for 100 DAMs between MBCT and CT.

**Figure 7:**
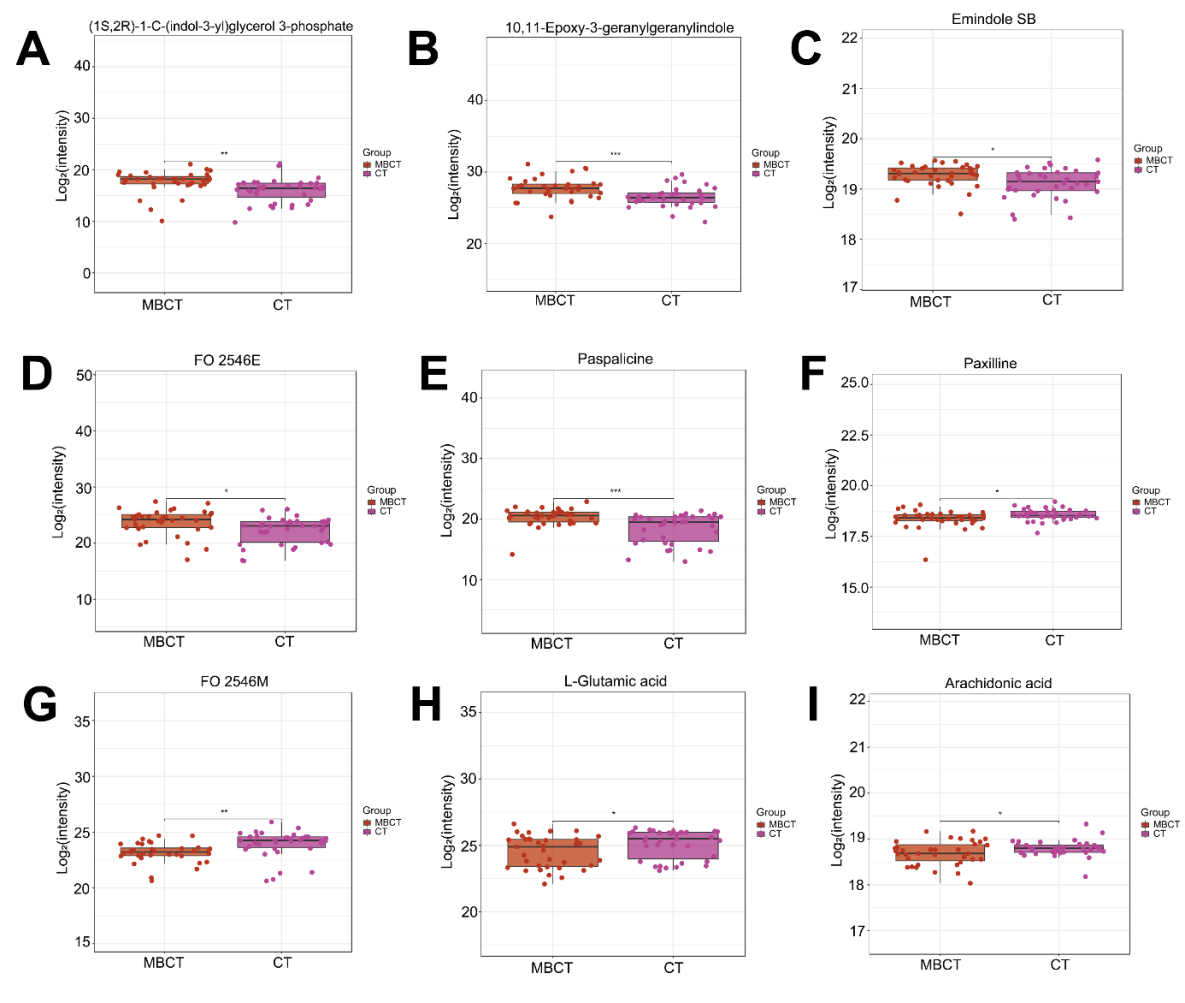
Normalized intensity of nine representative DAMs in 35 MBCT and 35 CT plasma samples. (A-E) The relative levels of five metabolites, including (1S, 2R)-1-C-(indol-3-yl) glycerol 3-phosphate, 10,11-epoxy-3-geranylgeranylindole, emindole SB, FO 2546E, and paspalicine, were significantly increased in MBCT compared to CT, while (F-I) paxilline, FO 2546M, L-glutamic acid, and arachidonic acid levels showed significant decreases. Statistical comparisons were performed using a T-test with BH method correction. *: Padj < 0.05, **: Padj < 0.01, ***: Padj < 0.001.

**Figure 8:**
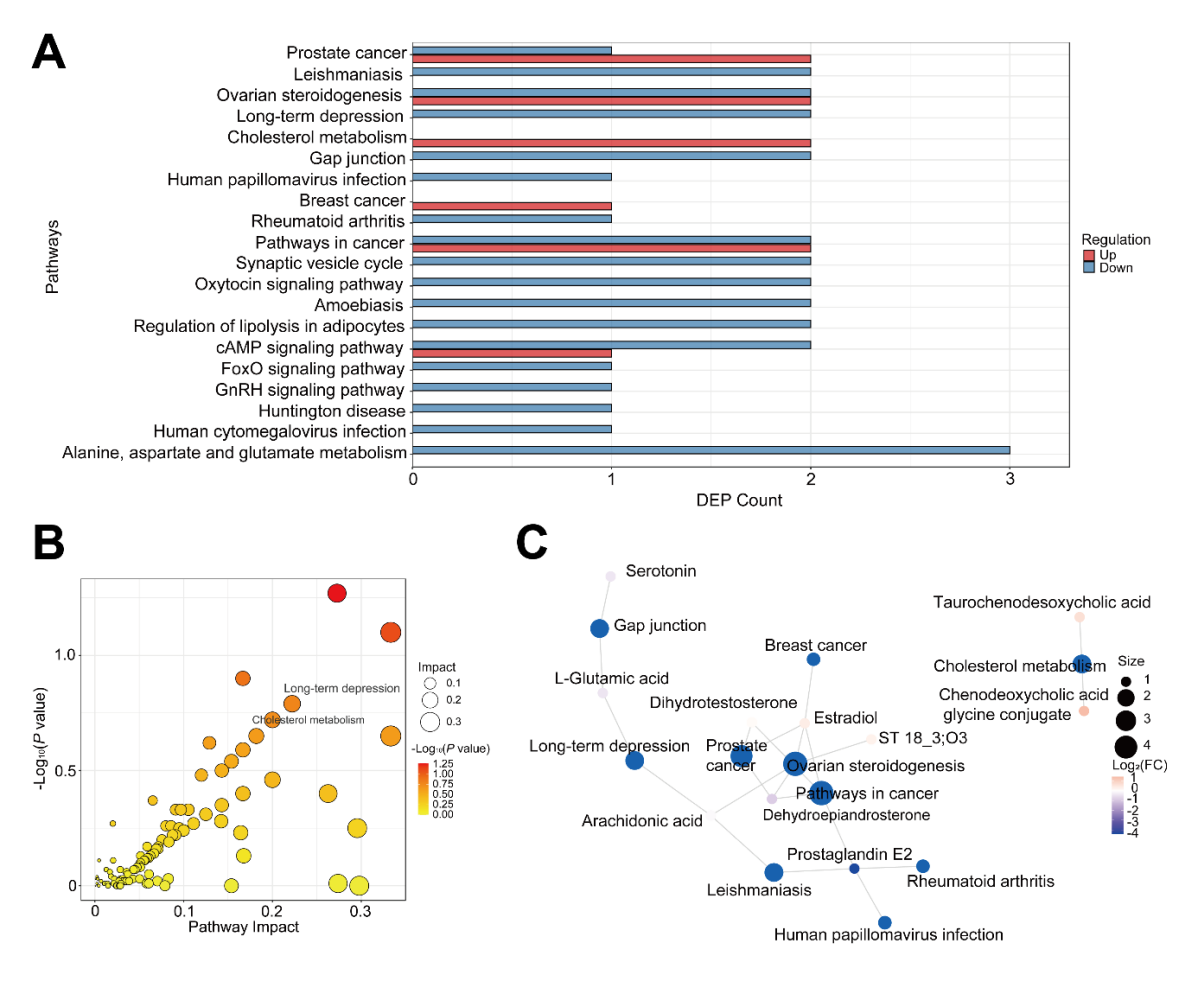
Functional and pathway enrichment analysis of 389 DAMs identified in MBCT vs. CT. (A) Top 20 ranking pathways enriched by up- and down-regulated DAMs. (B) Bubble plot showing long-term depression and cholesterol metabolism enriched by DAMs in MBCT vs. CT. (C) Interaction network analysis of representative DAMs and their participating pathways in MBCT.

To explore potential metabolite biomarkers related to MBCT's improvement of adolescent depression symptoms and the signaling pathways involved, we analyzed the C2 group. PLS-DA and OPLS-DA analyses in positive ion mode showed complete separation of MBCT and CT samples (Figures 5A-D(Fig. 5)), indicating homogeneity of samples within each group. Consistent results were observed in negative ion mode (Supplementary Figures 3A-D). Heatmap analysis revealed significant differences in the metabolic landscape of plasma samples between MBCT and CT groups (Figure 6A(Fig. 6)). Using VIP > 1.0 and P < 0.05, we identified 389 DAMs, including 203 up-regulated and 186 down-regulated metabolites, in the MBCT group compared to the CT group (Figures 6B and C(Fig. 6)). For example, 2-deoxy-D-glucose and (-)-bisdechlorogeodin levels significantly increased in the MBCT group (P < 0.05), while serylglycine, melanettin, and 9-hydroxyphenanthrene levels decreased (Figure 6D(Fig. 6)). Metabolites involved in long-term depression and indole diterpene alkaloid biosynthesis also showed significant changes. Specifically, (1S,2R)-1-C-(indol-3-yl) glycerol 3-phosphate, 10,11-epoxy-3-geranylgeranylindole, emindole SB, FO 2546E, and paspalicine levels increased significantly in the MBCT group compared to the CT group (P < 0.05) (Figures 7A-E(Fig. 7)). Whereas, paxilline, FO 2546M, L-glutamic acid, and arachidonic acid levels decreased in the MBCT group (Figures 7F-I(Fig. 7)). KEGG pathway enrichment analysis showed that down-regulated metabolites were mainly enriched in long-term depression, leishmaniasis, gap junction, human papillomavirus infection, synaptic vesicle cycle, oxytocin signaling, amoebiasis, regulation of lipolysis in adipocytes, FoxO signaling, GnRH signaling, Huntington disease, human cytomegalovirus infection, and alanine, aspartate and glutamate metabolism (Figure 8A(Fig. 8)). Up-regulated metabolites were primarily involved in prostate cancer, ovarian steroidogenesis, cholesterol metabolism, breast cancer, pathways in cancer, and cAMP signaling (Figure 8A(Fig. 8)). Notably, long-term depression and cholesterol metabolism were the top-ranking pathways (Figure 8B(Fig. 8)). Pathway interaction network analysis revealed that FO 2546E, FO 2546M, paxilline, paspalicine, and 14,15-epoxyemindole were associated with indole diterpene alkaloid biosynthesis, while taurochenodesoxycholic acid and chenodeoxycholic acid glycine conjugate directly related to cholesterol metabolism (Figure 8C(Fig. 8)). Arachidonic acid and L-glutamic acid were closely linked to long-term depression (Figure 8C(Fig. 8)).

### 3.4 The changes in differential metabolite concentrations are significantly correlated to MBCT improvement of clinical symptoms

**Figure 9:**
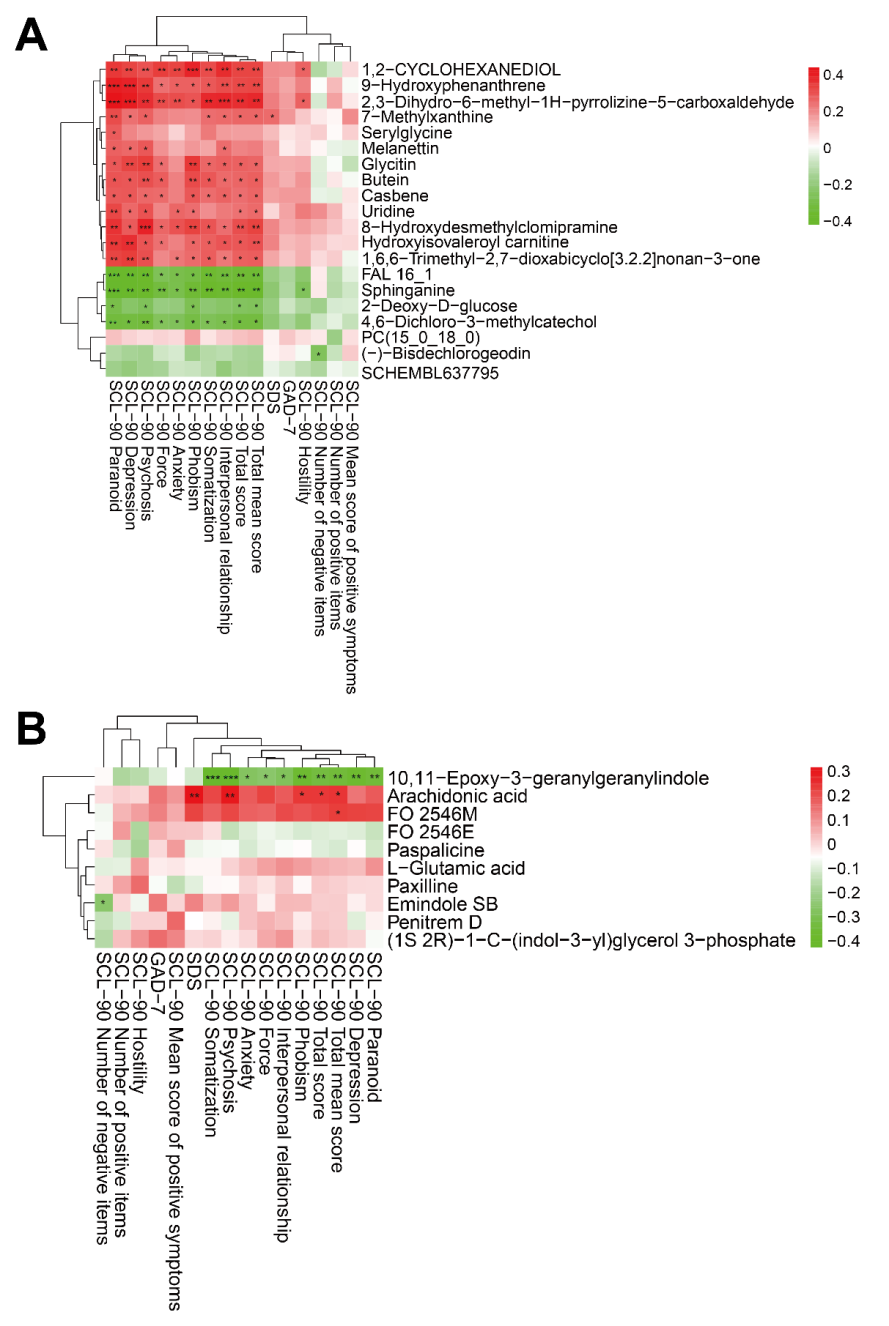
Significant correlations between altered levels of differential metabo-lites and MBCT improvement of clinical symptoms. (A) Pearson's correlation analysis showed extensive and significant correlation between level changes of top20 DAMs (such as melanettin and sphinganine) and MBCT improvement of clinical symp-toms, including SDS, GAD-7, and SCL-90 scores. (B) Pearson's correlation analysis represented significant correlation between metabolites involved in long-term depres-sion (such as arachidonic acid) and clinical symptoms score (SDS, GAD-7, and SCL-90). *: Padj < 0.05, **: Padj < 0.01, ***: Padj < 0.001.

To investigate whether these differential metabolites, especially those related to the long-term depression, are closely related to the improvement of clinical symptoms in MBCT group, we further performed Pearson's correlation analysis. The results showed that the most significant 20 DAMs were widely and significantly correlated with the improvement of clinical symptoms in MBCT group (Figure 9A(Fig. 9)). For example, the decrease in 7-methylxanthine concentration was significantly positively correlated with the improvement of SDS score (r = 0.2596, adjusted P = 0.0300), and was also significantly positively correlated with the decrease in SCL-90 scores, including total score (r = 0.2678, adjusted P = 0.0250), total mean score (r = 0.2684, adjusted P = 0.0247), and the score of interpersonal relationship (r = 0.2611, adjusted P = 0.0290), psychosis (r = 0.2750, adjusted P = 0.0212), depression (r = 0.2483, adjusted P = 0.0382), and paranoid (r = 0.3192, adjusted P = 0.0071) (Figure 9A(Fig. 9)). While the decrease in glycitin level was significantly positively correlated with the improvement in SCL-90 scale total score (r = 0.2776, adjusted P = 0.0200), total mean score (r = 0.2718, adjusted P = 0.0228), interpersonal relationship (r = 0.2833, adjusted P = 0.0175), phobism (r = 0.3600, adjusted P = 0.0022), force (r = 0.2495, adjusted P = 0.0373), psychosis (r = 0.3516, adjusted P = 0.0028), and depression (r = 0.3337, adjusted P = 0.0048) scores (Figure 9A(Fig. 9)), respectively. Similarly, the decrease in the content of melanettin, butein and casbene was also significantly positively correlated with the improvement of SCL-90 score (adjusted P < 0.05) (Figure 9A(Fig. 9)). Conversely, the increase in sphinganine concentration was significantly negatively correlated with the improvement in SCL-90 scores, including total score (r = -0.3597, adjusted P = 0.0022), total mean score (r = -0.3687, adjusted P = 0.0017), and the score of depression (r = -0.3844, adjusted P = 0.0010), anxiety (r = -0.2743, adjusted P = 0.0216), paranoid (r = -0.4130, adjusted P = 0.0004), psychosis (r = -0.3695, adjusted P = 0.0016), force (r = -0.3090, adjusted P = 0.0092), phobism (r = -0.3281, adjusted P = 0.0056), somatization (r = -0.3707, adjusted P = 0.0016), interpersonal relationship (r = -0.3470, adjusted P = 0.0033), and hostility (r = -0.2480, adjusted P = 0.0385) (Figure 9A(Fig. 9)). It is worth noting that the changes in the concentration of metabolites involved in long-term depression pathway were significantly correlated with the improvement of clinical symptoms in MBCT group. For instance, the decrease in arachidonic acid concentration was significantly positively correlated with the improvement of SDS and SCL-90 scores (including total score, total mean score, phobism and psychosis) in MBCT group (adjusted P < 0.05), respectively (Figure 9B(Fig. 9)). Specifically, the decrease in arachidonic acid concentration was significantly positively correlated with the decrease in SDS score (r = 0.3239, adjusted P = 0.0062), and was also significantly positively correlated with the decrease in SCL-90 scores, including total score (r = 0.2448, adjusted P = 0.0411), total mean score (r = 0.2502, adjusted P = 0.0367), phobism score (r = 0.2483, adjusted P = 0.0382), and psychosis score (r = 0.3075, adjusted P = 0.0096) (Figure 9B(Fig. 9)).

**Figure 10:**
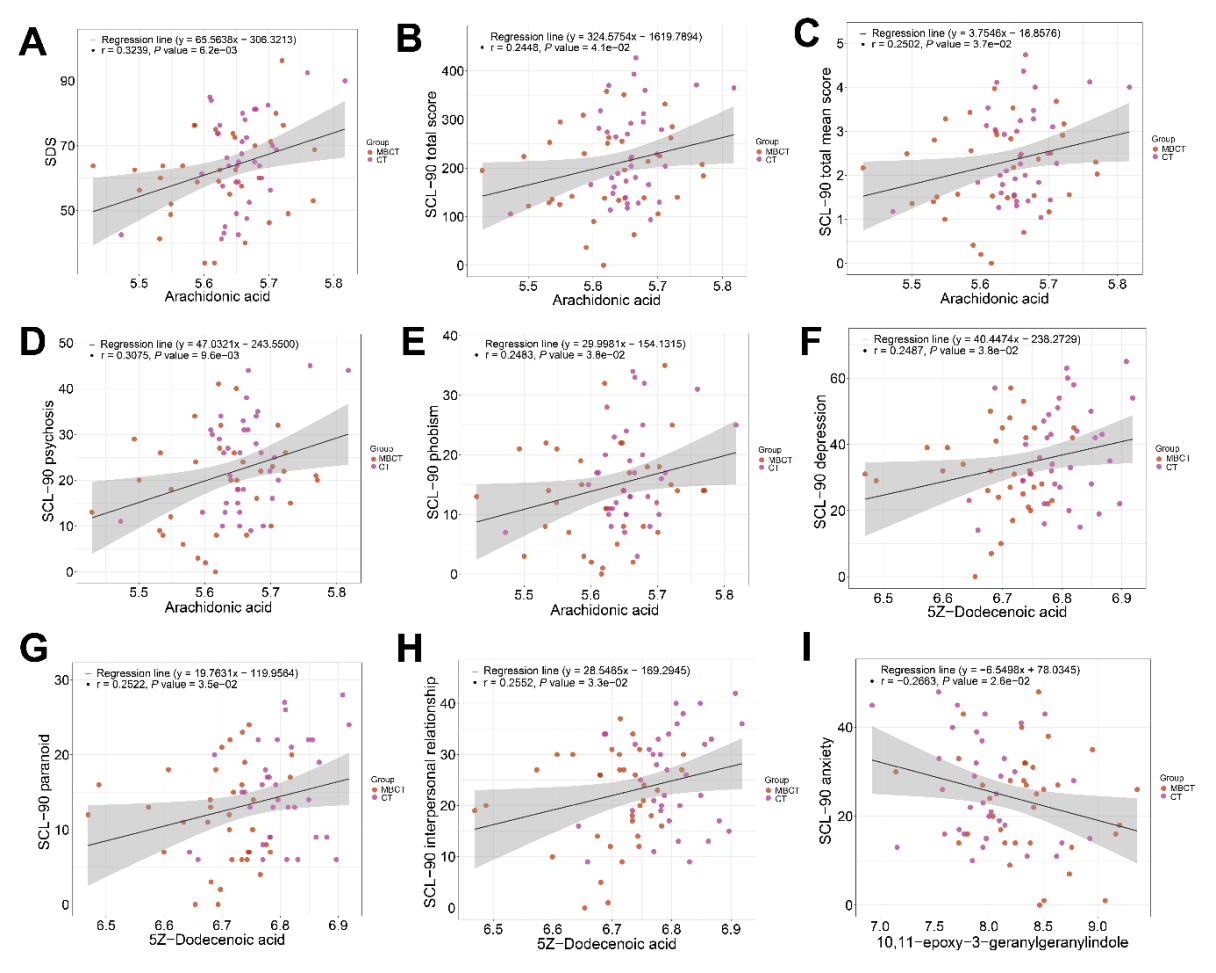
Level changes of arachidonic acid and 5Z-dodecenoic acid significantly correlated with the improvement of clinical scores. Significant and positive correlation between decreased level of arachidonic acid and reduced SDS score, SCL-90 total score, SCL-90 total mean score, SCL-90 psychosis score, and SCL-90 phobism score (A-E). Significant and positive correlation between decreased level of 5Z-dodecenoic acid and reduced SCL-90 depression score, SCL-90 paranoid score, and SCL-90 interpersonal relationship score (F-H). Significant and negative correlation between increased level of 10,11-epoxy-3-geranylgeranylindole and reduced SCL-90 anxiety score (I). Linear regression model was used to determine the correlation coefficient (r) and probability (P). The gray area around the straight line denoted 95 % confidence interval.

Consistently, linear regression analysis showed that the decrease in arachidonic acid concentration was significantly positively correlated with the decrease in SDS score (r = 0.3239, P = 0.0062) (Figure 10A(Fig. 10)), and was also significantly positively correlated with the decrease in SCL-90 scale total score (r = 0.2448, P = 0.0410), total mean score (r = 0.2502, P = 0.0370), psychosis score (r = 0.3075, P = 0.0096) and phobism score (r = 0.2483, P = 0.0380) (Figures 10B-E(Fig. 10)), respectively. In addition, the decrease in 5Z-dodecenoic acid content was significantly positively correlated with the reduction in SCL90 scale depression score (r = 0.2487, P = 0.0380), paranoid score (r = 0.2522, P = 0.0350), and interpersonal relationship score (r = 0.2552, P = 0.0330) (Figures 10F-H(Fig. 10)), respectively. While the upregulation in 10,11-epoxy-3-geranylgeranylindole abundance was significantly negatively correlated with reduced SCL-90 anxiety score (r = -0.2663, P = 0.0026) (Figure 10I(Fig. 10)). Taken together, these results indicated that the changes in metabolite concentrations in MBCT group were significantly correlated with the improvement of clinical symptoms, suggesting that certain metabolite such as arachidonic acid was likely involved in MBCT improvement of clinical symptoms in adolescent depression.

### 3.5 Potential biomarkers for evaluating MBCT efficacy and prognosis

**Figure 11:**
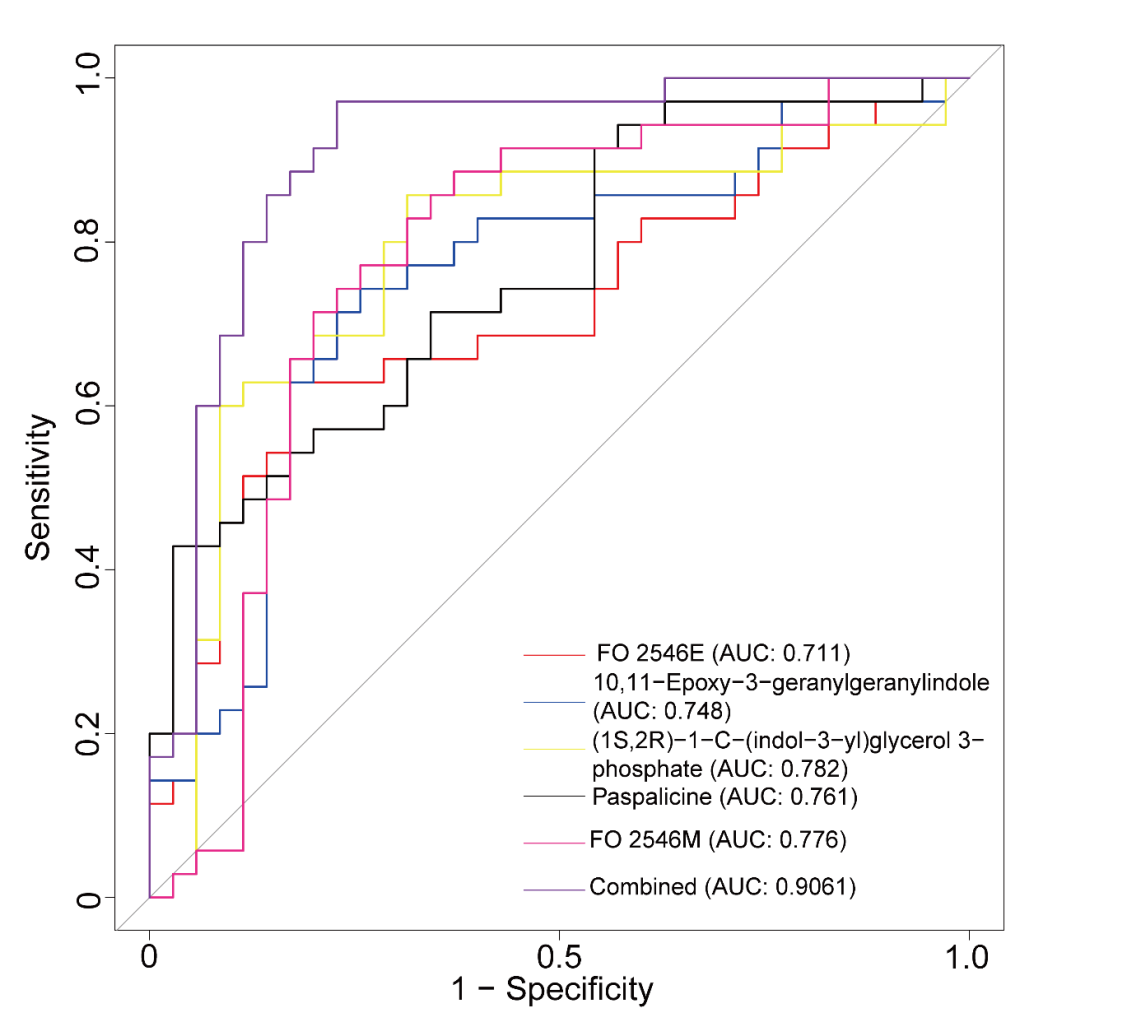
Receiver operating characteristic curves analysis of several DAMs for predicting MBCT efficacy. Receiver operating characteristic curves (ROC) for five DAMs, including FO 2546E, 10,11-epoxy-3-geranylgeranylindole, (1S, 2R)-1-C-(indol-3-yl) glycerol 3-phosphate, paspalicine, and FO 2546M, and the combination of these five DAMs. ROC analysis was performed using the pROC package (v.1.18.0) in R.

To identify potential biomarkers for assessing the efficacy and prognosis of MBCT in adolescent depression, receiver operating characteristic analysis was performed. Results showed that several metabolites involved in indole diterpene alkaloid biosynthesis exhibited promising potential for predicting MBCT efficacy. Specifically, FO 2546E (AUC = 0.711, sensitivity = 82.90 %, specificity = 62.90 %, 95 % CI: 0.587-0.835), 10,11-epoxy-3-geranylgeranylindole (AUC = 0.748, sensitivity = 77.10 %, specificity = 71.40 %, 95 % CI: 0.628-0.867), (1S,2R)-1-C-(indol-3-yl) glycerol 3-phosphate (AUC = 0.782, sensitivity = 68.60 %, specificity = 85.70 %, 95 % CI: 0.665-0.899), paspalicine (AUC = 0.761, sensitivity = 97.10 %, specificity = 42.90 %, 95 % CI: 0.649-0.872), and FO 2546M (AUC = 0.776, sensitivity = 80.00 %, specificity = 71.40 %, 95 % CI: 0.657-0.894) showed significant predictive potential (Figure 11(Fig. 11)). Notably, the combination of these five metabolites demonstrated high potential for evaluating the effect of MBCT intervention, with an AUC value of 0.9061 (sensitivity = 77.10 %, specificity = 97.1 %, 95 % CI: 0.830-0.982) (Figure 11(Fig. 11)), suggesting that this combination may serve as an auxiliary marker for predicting MBCT efficacy. Moreover, several metabolites involved in long-term depression, synaptic vesicle cycle, and cholesterol metabolism also showed potential in predicting MBCT efficacy. For instance, L-glutamic acid (AUC = 0.649, sensitivity = 51.40 %, specificity = 80.00 %, 95 % CI: 0.519-0.779), arachidonic acid (AUC = 0.635, sensitivity = 82.90 %, specificity = 51.40 %, 95 % CI: 0.497-0.773), serotonin (AUC = 0.642, sensitivity = 68.60 %, specificity = 65.70 %, 95 % CI: 0.510-0.773), taurochenodesoxycholic acid (AUC = 0.751, sensitivity = 68.60 %, specificity = 80.00 %, 95 % CI: 0.634-0.868), and chenodeoxycholic acid glycine conjugate (AUC = 0.684, sensitivity = 82.90 %, specificity = 51.40 %, 95 % CI: 0.558-0.810) (Supplementary Figure 4). Although individual metabolite indicators showed low specificity and sensitivity, the combination of the above five metabolites demonstrated high potential for predicting MBCT efficacy (AUC = 0.820, sensitivity = 82.90 %, specificity = 71.40 %, 95 % CI: 0.721-0.920) (Supplementary Figure 4).

Additionally, we observed that the relative levels of some metabolites were significantly down-regulated in depression-affected patients in the CT group compared to HC (adjusted P < 0.05), such as deoxyribose 1-phosphate, ganolucidic acid A, 3-dehydro-D-glucose 6-phosphate, (R)-carvone, 3-hydroxy-2-naphthoate, and 5-hydroxyxanthotoxin (Supplementary Figures 5A-F). In contrast, the relative concentrations of these six metabolites were significantly up-regulated in depression patients receiving MBCT compared to those in the CT group (adjusted P < 0.05) (Supplementary Figures 5A-F). Furthermore, the relative abundances of 8-hydroxydesmethylclomipramine, albendazole, and dihydrobisanhydro-bacterioruberin were significantly up-regulated in depression patients in the CT group compared to HC (adjusted P < 0.05) (Supplementary Figures 5G-I). However, the relative contents of these three metabolites were markedly down-regulated in depression patients receiving MBCT compared to CT (Supplementary Figures 5G-I). These results suggest that these metabolites and their associated pathways may be involved in MBCT's improvement of depression symptoms in adolescents.

## 4 Discussion

The increasing prevalence of depression, especially among adolescents, poses a significant global health challenge. Depression is a leading cause of disability worldwide (Thapar et al., 2012[119], Auerbach et al., 2016[6], Diener et al., 2021[25], Hazell, 2021[47], Thapar et al., 2022[120]). Depression arises from a combination of genetic, neurobiological, environmental, and psychological factors (Malhi and Mann, 2018[71]). Adolescents are highly vulnerable to depression due to dynamic developmental changes. Current treatments, including pharmacotherapy and psychotherapy, face challenges like variable efficacy, side effects, and low adherence. The complex pathological mechanisms of depression complicate diagnosis, treatment, and prognosis, presenting significant clinical challenges.

MBCT has been recognized as an effective intervention for various mental health conditions, particularly depression (Kuyken et al., 2015[60], Alsubaie et al., 2017[2], Cladder-Micus et al., 2018[17], Goldberg et al., 2019[39], Segal et al., 2020[103], Boge et al., 2021[10], Ritvo et al., 2021[96], Spinhoven et al., 2022[110], Hanssen et al., 2023[45], Dai et al., 2024[19], Hanssen et al., 2024[46]). MBCT integrates cognitive behavioral techniques with mindfulness strategies, promoting non-judgmental awareness of thoughts and feelings. This approach reduces relapse rates in recurrent depression and improves overall mental health outcomes (Williams et al., 2014[132]). Recent research highlights mindfulness-based intervention's potential to modulate metabolic and immune-inflammatory pathways, contributing to their therapeutic effects (Andres-Rodriguez et al., 2019[3]). Studies show mindfulness practices influence pro-inflammatory markers and improve metabolic health, which are critical in depression's pathophysiology (Marinovic and Hunter, 2022[72]). Our study shows MBCT significantly improved anxiety and depression symptoms in adolescents, with GAD-7 and SDS scores demonstrating P < 0.0001 (Table 2(Tab. 2)). Conversely, CT showed no significant improvement in anxiety symptoms (Table 2(Tab. 2)). SCL-90 scores indicate MBCT significantly improved psychological symptoms (P < 0.05) and alleviated positive symptoms in adolescent patients (P < 0.05) (Table 3(Tab. 3)). Despite these advancements, the specific metabolic mechanisms underlying MBCT's alleviation of depressive symptoms remain unclear.

Research is urgently needed to clarify these mechanisms and identify reliable, specific, and sensitive biomarkers to evaluate MBCT efficacy and predict treatment outcomes. L-Glutamine (L-Glu) plays a crucial role in physiological processes including inflammatory response, oxidative stress, and neuronal activity regulation (Niihara et al., 2018[83], Gwangwa et al., 2019[44], Ogando et al., 2019[84], Mates et al., 2020[75], Petrus et al., 2020[87], Starling, 2020[111], de Oliveira Santos et al., 2021[23], Jones et al., 2021[56], Arra et al., 2022[5], Cheung et al., 2022[15], Kim et al., 2022[59], Thomas et al., 2022[122], Baek et al., 2023[7], Sun et al., 2023[114], Jin et al., 2024[55], Leite et al., 2025[65]). As the primary excitatory neurotransmitter in the central nervous system (CNS), L-Glu is involved in synaptic transmission, learning, and memory (Somers et al., 1982[107], Fricke et al., 2007[32], Gibbs et al., 2008[38], Valladolid-Acebes et al., 2012[124], Thielen et al., 2018[121], Xin et al., 2019[137], Cheung et al., 2022[15]). L-Glu is also a precursor for synthesizing other important molecules like γ-aminobutyric acid (GABA), the primary inhibitory neurotransmitter in the CNS. In depression, L-Glu is implicated in the disorder's pathophysiology. Studies show alterations in glutamate levels and glutamatergic signaling are associated with depressive symptoms (de Lima et al., 2009[22], Yoon et al., 2009[142], Moriguchi et al., 2019[80], Kantrowitz et al., 2021[57]). For example, altered L-Glu levels in the prefrontal cortex are observed in animal depression models, contributing to cognitive and emotional deficits (Lee et al., 2013[63], Veeraiah et al., 2014[126], Pu et al., 2021[89]). Moreover, L-Glu signaling via the N-methyl-D-aspartate receptor (NMDAR) is linked to synaptic plasticity and stress resilience, which are critical in depression's development and treatment (Guo et al., 2012[43], Bodner et al., 2020[9]). Our study shows plasma L-glutamic acid levels were significantly downregulated in the MBCT group compared to the CT group (P < 0.05) (Figures 7H and I(Fig. 7)). Moreover, long-term depression, a signaling pathway involving L-glutamic acid, was identified as one of the top-ranking pathways (Figures 8A-C(Fig. 8)). Our findings also showed that plasma L-Glu levels were significantly lower in APWD than in HCs (P < 0.001) (Figure 3A(Fig. 3)), and GABAergic synapse regulation ranked highly among metabolic pathways (Figure 4A(Fig. 4)). These results indicate MBCT may alleviate depressive and anxiety symptoms by modulating L-Glu-related inflammatory responses, oxidative stress, and neuronal activity.

Arachidonic acid (AA), an n-6 polyunsaturated fatty acid, serves as a precursor for synthesizing bioactive lipid mediators like prostaglandins, leukotrienes, and endocannabinoids. These eicosanoids are involved in physiological processes including inflammation and immune responses (Brain and Williams, 1990[13], Funk, 2001[33], Ricciotti and FitzGerald, 2011[94], Aoki and Narumiya, 2012[4], Martinez-Colon and Moore, 2018[73], Maseda et al., 2019[74], McGinty et al., 2020[77], Rahaman and Ganguly, 2021[90], Xin et al., 2024[138], Lee et al., 2025[62]). Studies suggest AA and its product eicosanoids, such as prostaglandin E2 (PGE2), may contribute to depression's pathogenesis via neuroinflammation and oxidative stress (Song et al., 2018[108], Song et al., 2019[109], Saliba et al., 2021[100]). Dysregulated AA metabolism is linked to several neuropsychiatric disorders, including depression (Feinmark et al., 2003[29], Yui et al., 2015[143]). Previous studies show AA levels are significantly elevated in depressed patients and animal models (Green et al., 2005[41]), potentially serving as an auxiliary diagnostic biomarker (Gao et al., 2021[35]). Early research indicates AA may act as a potential retrograde messenger in hippocampal long-term depression (Bolshakov and Siegelbaum, 1995[11]). A cross-sectional study reports a higher AA to omega-3 fatty acids ratio is associated with depression onset in patients undergoing IFN-α therapy (Lotrich et al., 2013[68]). Another study shows depressed patients have higher ratios of AA to docosahexaenoic acid and AA to eicosapentaenoic acid than controls (Frasure-Smith et al., 2004[31]). Another study indicates AA may influence depression's pathophysiology via serotonin uptake (Gopaldas et al., 2019[40]). Our study shows circulating AA levels were significantly reduced in the MBCT group compared to the CT group (P < 0.05) (Figure 7I(Fig. 7)), suggesting MBCT downregulates AA concentrations. Furthermore, Pearson's correlation analysis showed that the decreased abundance of AA was significantly and positively correlated with the reduction of SDS score, SCL-90 total score, total mean score, psychosis, and phobism score (adjusted P < 0.05) (Figure 9B(Fig. 9)). The results of linear regression analysis further demonstrated the significant correlation between the changes in AA concentration and the improvement of these clinical symptoms scores (Figures 10A-E(Fig. 10)). These results imply reduced AA levels may contribute to MBCT's therapeutic effects on anxiety and depression by reducing neuroinflammation and oxidative stress in adolescents. Interestingly, metabolites related to indole diterpene alkaloid biosynthesis, such as 14,15-epoxyemindole, were unexpectedly observed in human plasma, likely originating from dietary sources or gut microbiota (Sinha et al., 2024[106]). Future research will further validate potential biomarkers like L-glutamic acid and arachidonic acid and investigate strategies to incorporate them into personalized treatment plans and treatment response monitoring. For example, using LC-MS/MS to measure the baseline levels and dynamic changes of specific plasma biomarkers (e.g., arachidonic acid) can help clinicians predict which patients may respond well to MBCT and identify those needing additional or alternative therapies. Dynamic monitoring of these plasma biomarkers enhances clinicians' ability to evaluate MBCT efficacy, predict prognosis, and adjust treatment plans in real-time.

Recent studies highlight inflammation's substantial role in depression, with proinflammatory cytokines and hypothalamic-pituitary-adrenal (HPA) axis dysfunction as key contributors (Walsh et al., 2016[127], Sanada et al., 2020[101], Marinovic and Hunter, 2022[72]). The pathological processes of chronic stress-related diseases like depression are primarily mediated via HPA axis and sympathetic-adrenal-medullary system activation. One study shows mindfulness practices, including socio-affective and socio-cognitive training, significantly dampen physiological stress responses, particularly HPA axis end-product cortisol production, with reductions up to 51 % (Engert et al., 2017[26]). Lower cortisol levels may reduce stress-related metabolite production, such as L-glutamic acid and arachidonic acid, promoting a more balanced metabolic state. Furthermore, mindfulness-based interventions for depression and anxiety reduce inflammation and oxidative stress, which are closely linked to metabolic alterations (Bower et al., 2015[12], Walsh et al., 2016[127], Kenne Sarenmalm et al., 2017[58], Hoge et al., 2018[48], Sanada et al., 2020[101]). For instance, Pascoe et al. demonstrated mindfulness meditation was associated with decreased cortisol, C-reactive protein (CRP), triglycerides, and proinflammatory cytokines like tumor necrosis factor-α (TNF-α) (Pascoe et al., 2017[86]). Thus, MBCT may significantly reshape the metabolic profile by reducing inflammatory responses and modulating HPA-cortisol signaling.

However, the current study has several limitations. First, participants, including adolescents with depression and healthy controls, were recruited from a single center with a relatively small sample size, increasing the risk of false-positive results. Future studies should recruit larger, multi-center cohorts to validate these findings and further establish the clinical relevance of candidate biomarkers (e.g., arachidonic acid) and MBCT's role in alleviating anxiety and depression. Second, while this study identified candidate biomarkers and signaling pathways related to MBCT's improvement of depressive and anxiety symptoms in adolescents, further validation in different age groups (e.g., adults with depression) is needed. Additionally, to determine whether these potential biomarkers associated with MBCT's therapeutic efficacy are specific to adolescent depression, future validation in different disorders like anxiety and post-traumatic stress disorder is planned. Third, the relatively short-term MBCT intervention limits our ability to assess its long-term efficacy. Based on cross-sectional plasma metabolomic profiling, metabolic landscapes and differential metabolite changes associated with MBCT were observed. Future studies will investigate the relationship between metabolic shifts and long-term MBCT outcomes to determine whether target metabolites (e.g., arachidonic acid) are mechanistically involved in MBCT's improvement of depressive symptoms and how their concentration changes correlate with sustained MBCT effects over time. Fourth, while this study strictly controlled for age, sex, and batch effects on metabolomic data, other potential confounding factors like diet, exercise, and gut microbiota, may still influence the metabolic profile. Dietary patterns can alter amino acid and fatty acid concentrations, exercise can increase metabolite levels like lactic acid and pyruvic acid, and gut microbiota can synthesize metabolites such as propionic acid and butyric acid. Future research will collect comprehensive data on participants' diet and exercise and conduct gut microbiome studies via metagenomic sequencing. This will enable a more precise assessment of how MBCT affects metabolic profiles and help identify potential biomarkers. Fifth, this study reports metabolomic findings from a small sample size, identifying MBCT-associated metabolite signatures and potentially participating signaling pathways. However, functional studies and mouse model experiments are needed to clarify the molecular mechanisms by which these metabolites (e.g., L-glutamic acid and arachidonic acid) and related pathways contribute to MBCT's therapeutic effects on depression and anxiety. Sixth, while peripheral metabolic profile changes and metabolite signatures following MBCT intervention were characterized, these findings provide limited insight into the molecular mechanisms underlying MBCT's impact on depressive symptoms. Future research will integrate multimodal neuroimaging (e.g., functional magnetic resonance imaging), brain organoid models, and single-cell transcriptomic sequencing to investigate MBCT's effects on neural activity and the regulatory mechanism of target metabolites in depression's neuropathology.

## 5 Conclusion

In conclusion, our study shows MBCT significantly reduces anxiety and depressive in adolescents, likely via metabolomic modulation. We characterized the plasma metabolic landscape in depressed adolescents and identified biomarkers and pathways linked to MBCT's effects. These findings provide candidate biomarkers and evidence for predicting MBCT intervention outcomes. This study advances our understanding of MBCT in depression treatment and highlights the need for further research to identify specific biomarkers and clarify molecular mechanisms. The research offers a promising strategy to enhance precision and efficacy in treating adolescent depression.

## Declaration

### Ethic approval

The protocols of this study were reviewed and approved by the Ethics Committee of Wuhu Fourth People's Hospital (Approval number [2022]--KY--14), and the Ethics Committee of Lishui Second People's Hospital (Approval number Lun Shen 2025 Yan Di (027) Hao) prior to the recruitment of subjects. All subjects provided their written informed consent to participate in this study.

### Conflict of interest statement

The authors declare that they have no conflict of interest.

### Funding

This study was funded by grants from the Medicine and Health Science and Technology Project of Zhejiang Province (No. 2025KY509), the Research Fund for Lin He Academician New Medicine (No. 22340003), and the Basic Research Program of Guizhou Province (No. QianKeHe Basics-ZK [2023] General 583). The funding body has no role in designing the study, sampling, and interpretation of data and in writing the manuscript.

### Author contributions

Conceptualization, C.H.X., F.Y.; Methodology, H.L., Q.P.W., G.Q.M., and D.J.Z.; Subject recruitment, B.L.Z., C.L.G., and L.W.; Formal analysis, C.H.X., S.C., and F.Y.; Writing-original draft preparation, F.Y.; Writing-review and editing, C.H.X.; Supervision, F.Y.; Project administration, F.Y. All authors have reviewed and agreed to the published version of the manuscript.

### Artificial Intelligence (AI) - assisted technology

None was used in any stage of this work.

### Data availability statement

All datasets generated and/or analyzed during the current study are available from the corresponding author upon reasonable request.

### Acknowledgment

We thank all recruited patients and healthy volunteers for participating in this study.

## Supplementary Material

Supplementary information

## Figures and Tables

**Table 1:**
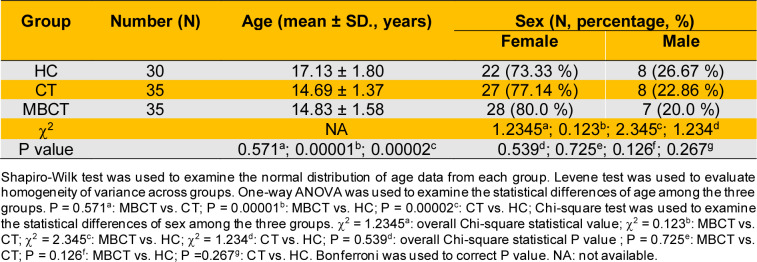
Characteristics of all participants

**Table 2:**
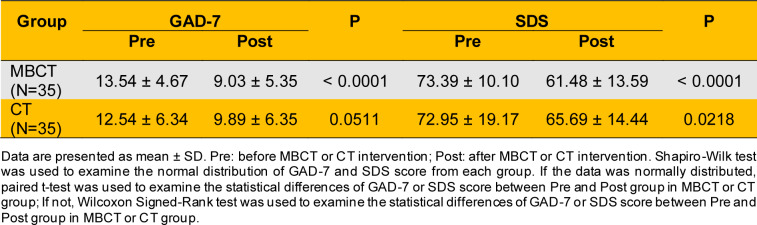
GAD-7 and SDS score of all patients with depression received MBCT or CT

**Table 3:**
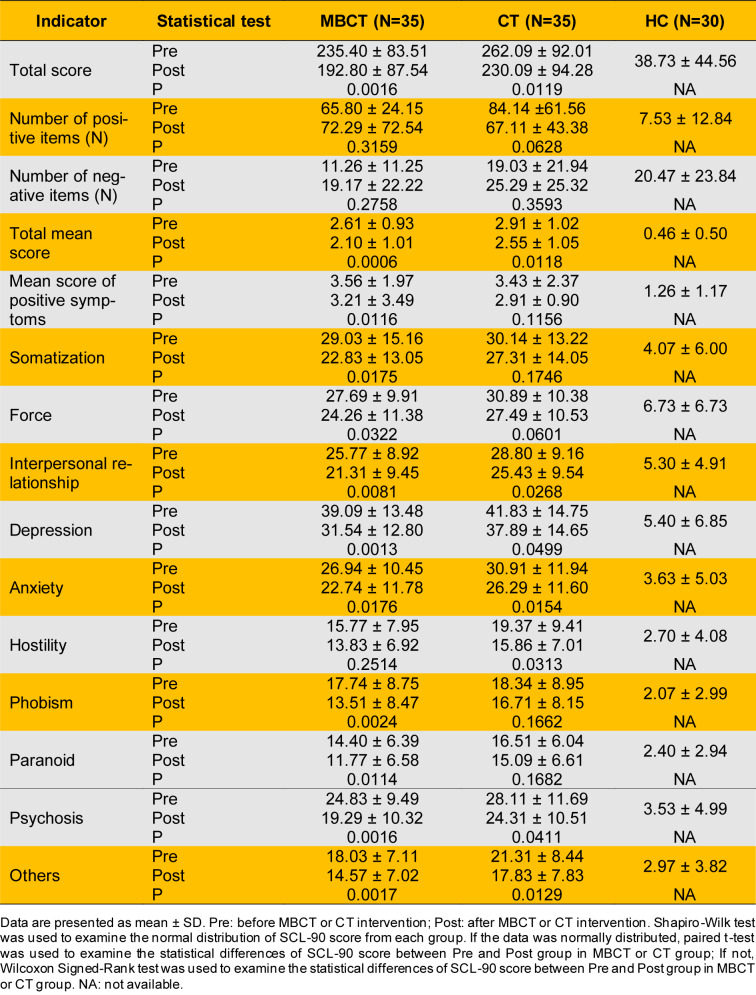
SCL-90 score of all patients with depression received MBCT or CT
