# Supplementary material for: Metabolic profiling reveals potential biomarkers and underlying signaling pathways involved in mindfulness-based cognitive therapy-improved adolescent depression symptoms
Source: EXCLI J. 2026 Jan 5;25:68–101. doi: 10.17179/excli2025-8918 (PMC12901960; doi:10.17179/excli2025-8918)
Supplement: Supplementary information [file EXCLI-25-68-s-001.pdf]

EXCLI Journal 2026;25:68-101 – ISSN 1611-2156
Received: September 12, 2025, accepted: October 17, 2025, published: January 05, 2026

Supplementary information to:

Original article:

METABOLIC PROFILING REVEALS POTENTIAL BIOMARKERS AND UNDERLYING SIGNALING PATHWAYS INVOLVED IN MINDFULNESS-BASED COGNITIVE THERAPY-IMPROVED ADOLESCENT DEPRESSION SYMPTOMS

Chun-Hua Xu[1], Bi-Lan Zhang[1], Chun-Lan Guan[2], Lin Wang[3], Shan Chao[4], He Li[5], Qiu-Ping Wu[6], Da-Jin Zhou[7], Guo-Qing Min[6], Fan Yang[5,8,*]

1 Department of Children and Adolescents, Anding Hospital Wuhu Hospital (Wuhu Fourth People's Hospital), Wuhu, China
2 Department of Nursing, Anding Hospital Wuhu Hospital (Wuhu Fourth People's Hospital), Wuhu, China
3 Department of Psychology, Anding Hospital Wuhu Hospital (Wuhu Fourth People's Hospital), Wuhu, China
4 The Research Center for Lin He Academician New Medicine, Institutes for Shanghai Pudong Decoding Life, Shanghai, China
5 Lishui Key Laboratory of Brain Health and Severe Brain Disorders, Lishui Second People's Hospital Affiliated to Wenzhou Medical University, Lishui, China
6 Department of Children and Adolescents, Lishui Second People's Hospital Affiliated to Wenzhou Medical University, Lishui, China
7 Department of Clinical Laboratory, Lishui Second People's Hospital Affiliated to Wenzhou Medical University, Lishui, China
8 Bio-X Institutes, Key Laboratory for the Genetics of Developmental and Neuropsychiatric Disorders, Ministry of Education, Shanghai Jiao Tong University, Shanghai, China

* **Corresponding author:** Fan Yang, PhD, Associate Investigator, No. 69, Beihuan Road, Liandu District, Lishui Key Laboratory of Brain Health and Severe Brain Disorders, Lishui Second People's Hospital Affiliated to Wenzhou Medical University, Lishui, 323000, Zhejiang Province, China; E-mail: yangfan@sibs.ac.cn

https://dx.doi.org/10.17179/excli2025-8918

This is an Open Access article distributed under the terms of the Creative Commons Attribution License (https://creativecommons.org/licenses/by/4.0/).

EXCLI Journal 2026;25:68-101 – ISSN 1611-2156
Received: September 12, 2025, accepted: October 17, 2025, published: January 05, 2026

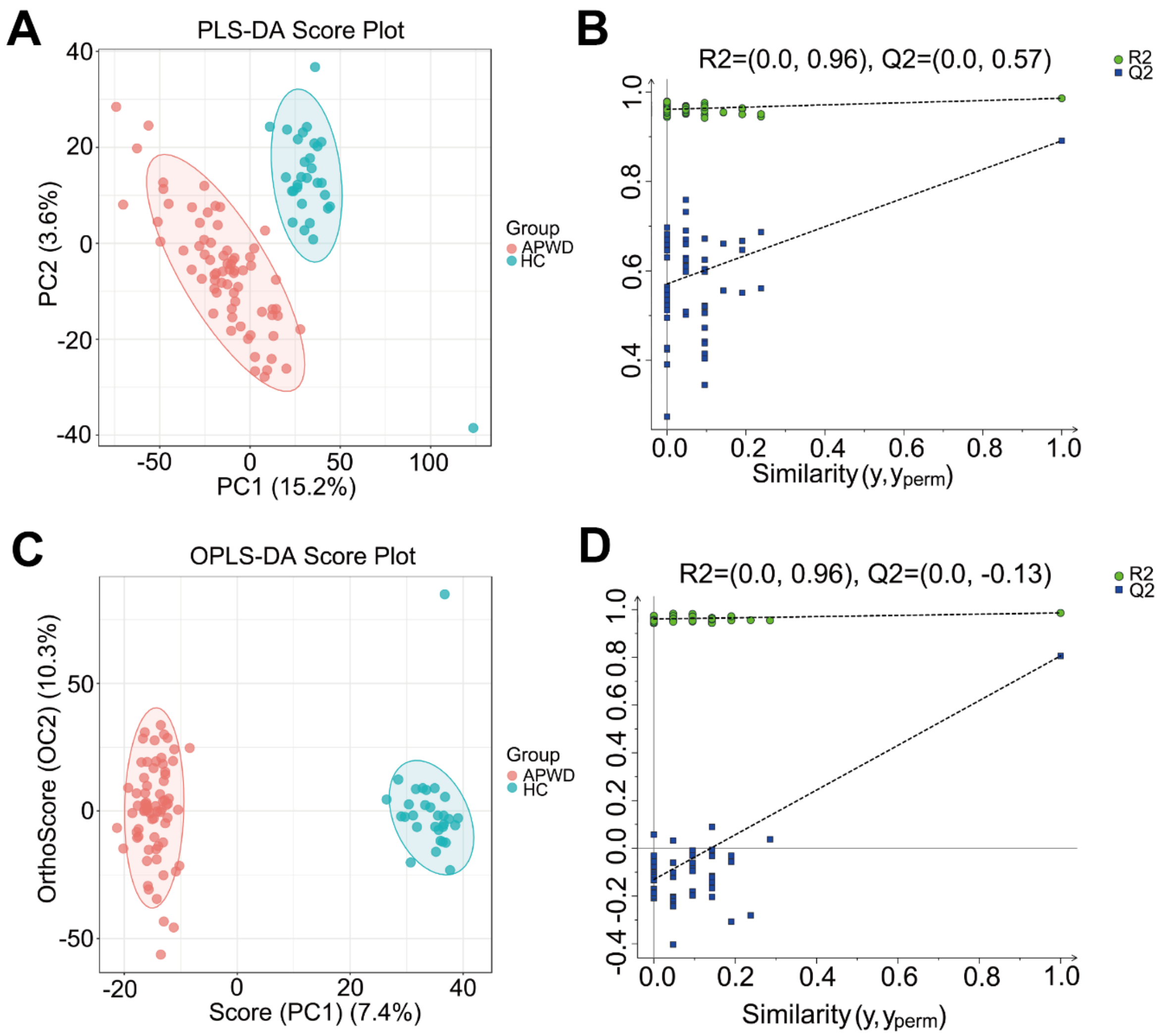

Supplementary Figure 1: PLS-DA and OPLS-DA models for separating APWD and HC in negative ion mode. (**A**) and (**B**) display the PLS-DA plot for the negative ion mode. R2 = (0.0, 0.96), Q2 = (0.0, 0.57). (**C**) and (**D**) present the OPLS-DA plot for the negative ion mode. R2 = (0.0, 0.96), Q2 = (0.0, -0.13).

Received: September 12, 2025, accepted: October 17, 2025, published: January 05, 2026

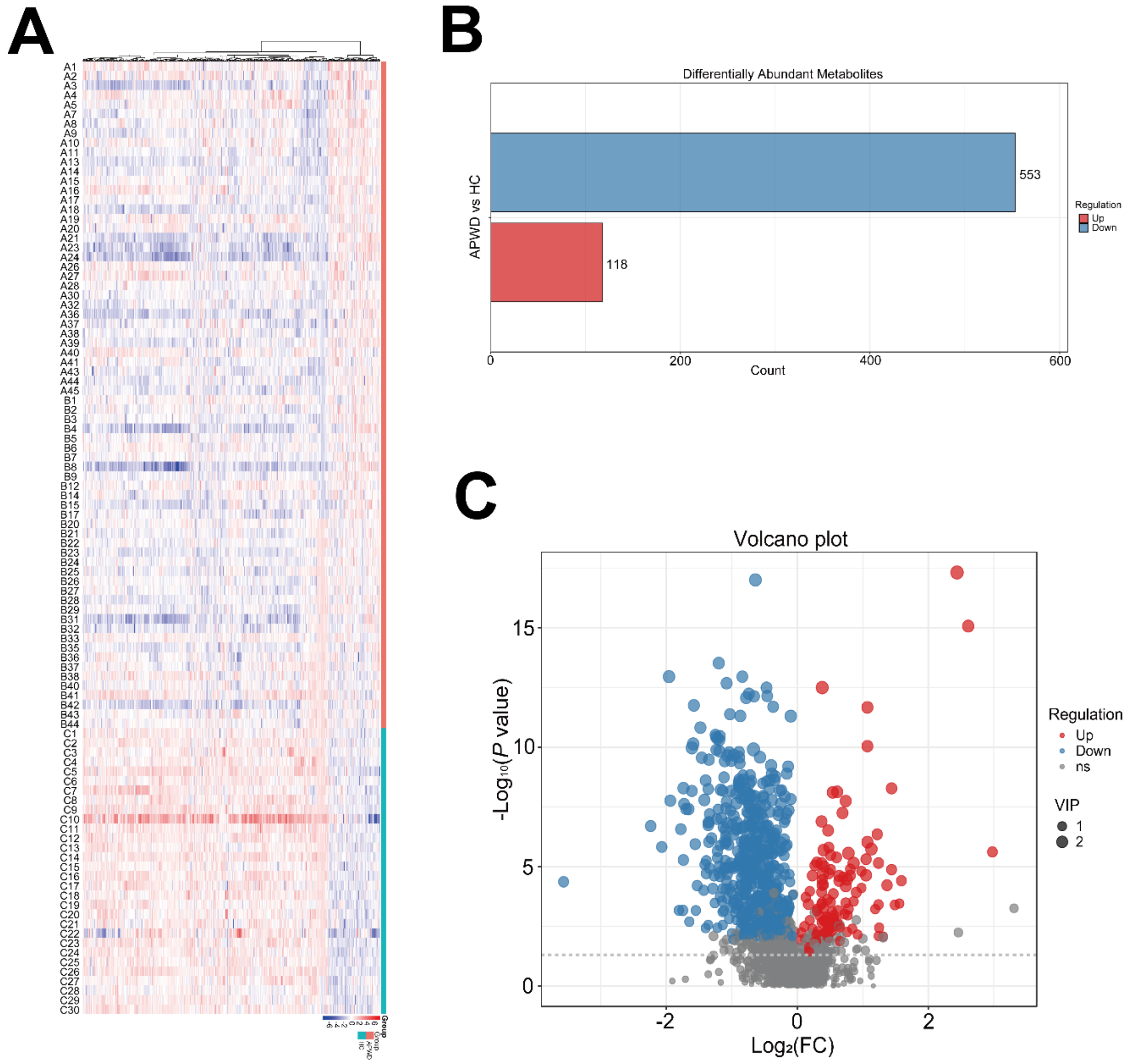

Supplementary Figure 2: Identification of 671 differential metabolites between APWD and HC. (**A**) The heatmap visualizes all DAMs, with blue and red indicating down- and up-regulation, respectively. Relative levels of metabolites were determined by the Pheatmap package (v.4.3.3) in R. (**B**) The bar plot and (**C**) the volcano plot shows the distribution of DAMs, including 118 up-regulated and 553 down-regulated metabolites in APWD.

EXCLI Journal 2026;25:68-101 – ISSN 1611-2156
Received: September 12, 2025, accepted: October 17, 2025, published: January 05, 2026

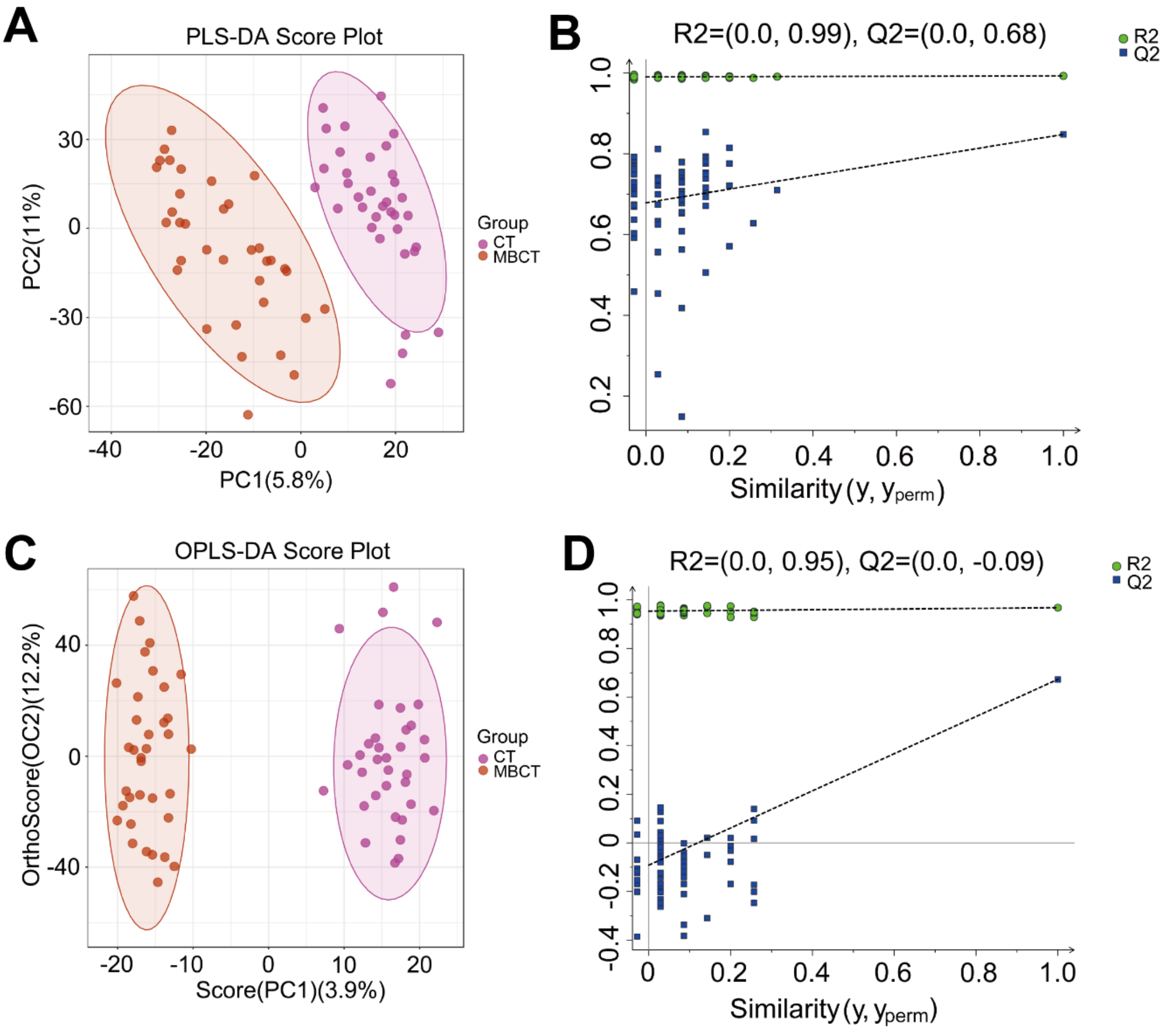

Supplementary Figure 3: PLS-DA and OPLS-DA models for separating MBCT and CT in negative ion mode. (**A**) and (**B**) display the PLS-DA plot for the negative ion mode. R2 = (0.0, 0.99), Q2 = (0.0, 0.68). (**C**) and (**D**) present the OPLS-DA plot for the negative ion mode. R2 = (0.0, 0.95), Q2 = (0.0, -0.09).

EXCLI Journal 2026;25:68-101 – ISSN 1611-2156
Received: September 12, 2025, accepted: October 17, 2025, published: January 05, 2026

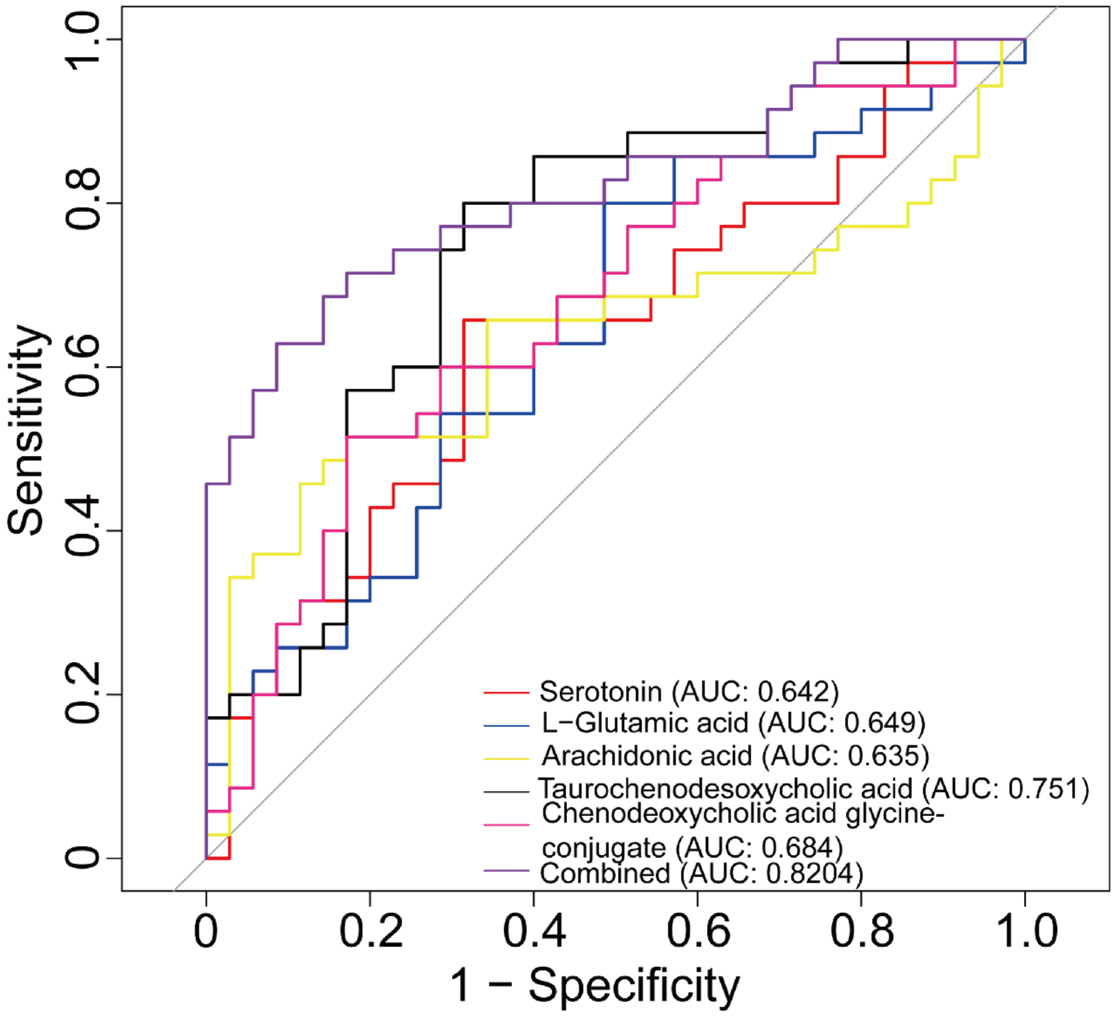

Supplementary Figure 4: Receiver operating characteristic analysis of five representative DAMs for predicting MBCT efficacy. Receiver operating characteristic (ROC) curves for five DAMs, including serotonin, L-glutamic acid, arachidonic acid, taurochenodesoxycholic acid, and chenodeoxycholic acid glycine conjugate, and the combination of these five DAMs. ROC analysis was performed using the pROC package (v.1.18.0) in R.

EXCLI Journal 2026;25:68-101 – ISSN 1611-2156
Received: September 12, 2025, accepted: October 17, 2025, published: January 05, 2026

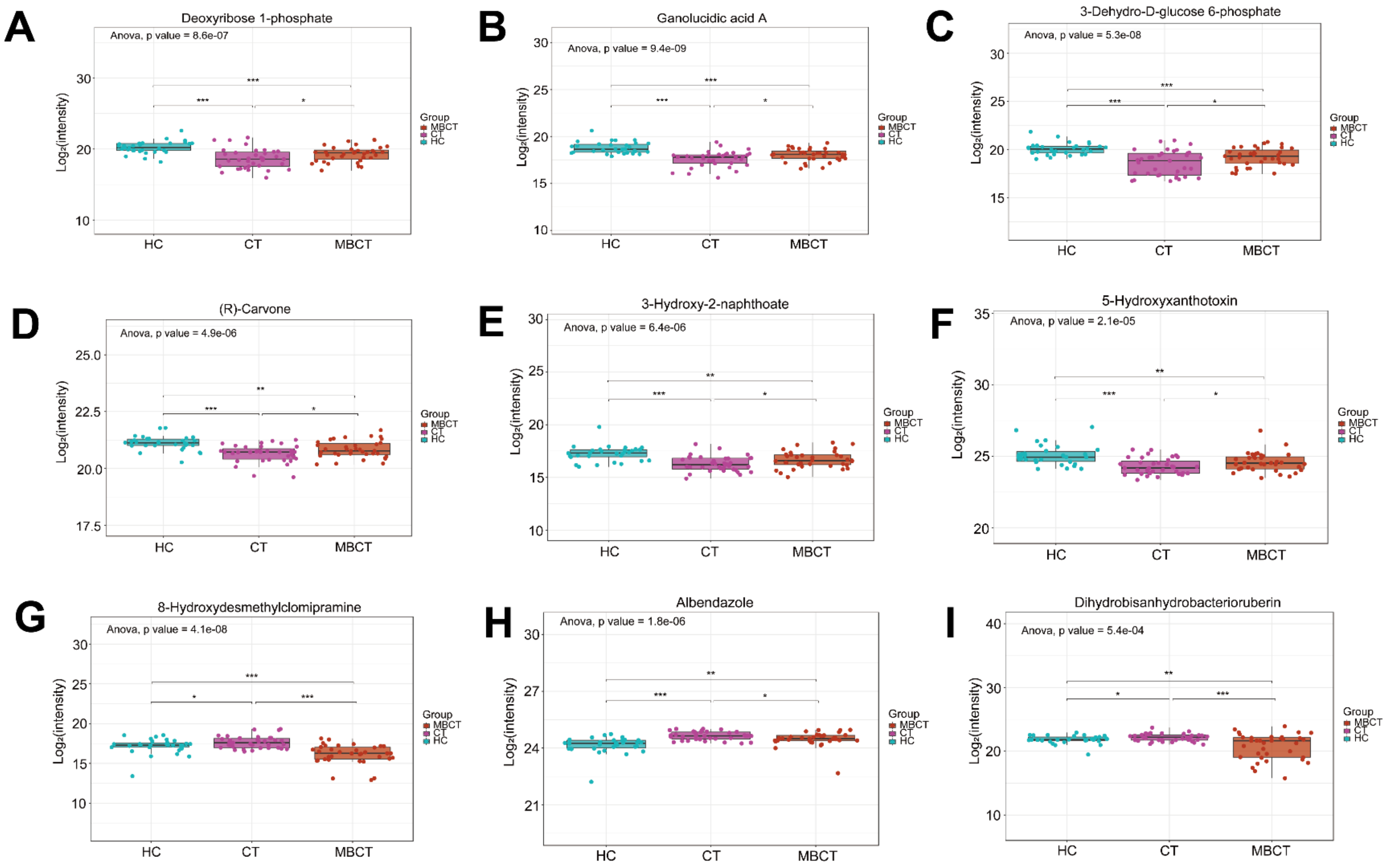

Supplementary Figure 5: Box plot of relative levels of differentially abundant metabolites in MBCT and CT. (**A-F**) Relative levels of six metabolites, including deoxyribose 1-phosphate, ganolucidic acid A, 3-dehydro-D-glucose 6-phosphate, (R)-carvone, 3-hydroxy-2-naphthoate, and 5-hydroxyxanthotoxin, were significantly down-regulated in CT compared to HC but up-regulated in MBCT relative to CT. (**G-I**) Relative levels of 8-hydroxydesmethylclomipramine, albendazole, and dihydrobisanhydrobacterioruberin were significantly up-regulated in CT compared to HC but down-regulated in MBCT relative to CT. Samples were compared using one-way ANOVA statistical analysis and Tukey's multiple comparisons post-hoc test. *: Padj < 0.05, **: Padj < 0.01, ***: Padj < 0.001.
